# Fatty acid biomarkers of dairy fat consumption and incidence of type 2 diabetes: A pooled analysis of prospective cohort studies

**DOI:** 10.1371/journal.pmed.1002670

**Published:** 2018-10-10

**Authors:** Fumiaki Imamura, Amanda Fretts, Matti Marklund, Andres V. Ardisson Korat, Wei-Sin Yang, Maria Lankinen, Waqas Qureshi, Catherine Helmer, Tzu-An Chen, Kerry Wong, Julie K. Bassett, Rachel Murphy, Nathan Tintle, Chaoyu Ian Yu, Ingeborg A. Brouwer, Kuo-Liong Chien, Alexis C. Frazier-Wood, Liana C. del Gobbo, Luc Djoussé, Johanna M. Geleijnse, Graham G. Giles, Janette de Goede, Vilmundur Gudnason, William S. Harris, Allison Hodge, Frank Hu, InterAct Consortium, Albert Koulman, Markku Laakso, Lars Lind, Hung-Ju Lin, Barbara McKnight, Kalina Rajaobelina, Ulf Risérus, Jennifer G. Robinson, Cécilia Samieri, David S. Siscovick, Sabita S. Soedamah-Muthu, Nona Sotoodehnia, Qi Sun, Michael Y. Tsai, Matti Uusitupa, Lynne E. Wagenknecht, Nick J. Wareham, Jason HY Wu, Renata Micha, Nita G. Forouhi, Rozenn N. Lemaitre, Dariush Mozaffarian, Fatty Acids and Outcomes Research Consortium (FORCE)

**Affiliations:** 1 MRC Epidemiology Unit, University of Cambridge School of Clinical Medicine, Cambridge, United Kingdom; 2 Cardiovascular Health Research Unit, Department of Medicine, University of Washington, Seattle, Washington, United States of America; 3 Department of Public Health and Caring Sciences, Clinical Nutrition and Metabolism, Uppsala University, Sweden; 4 Department of Nutrition and Epidemiology, Harvard T. H. Chan School of Public Health, Boston, Massachusetts, United States of America; 5 Institute of Epidemiology and Preventive Medicine, College of Public Health, National Taiwan University, Taipei City, Taiwan; 6 Institute of Public Health and Clinical Nutrition, University of Eastern Finland, Kuopio, Finland; 7 Section of Cardiovascular Medicine, Department of Internal Medicine, Wake Forest University School of Medicine, Bowman Gray Center, Winston-Salem, North Carolina, United States of America; 8 INSERM, UMR 1219, Bordeaux Population Health Research Center, University of Bordeaux, Bordeaux, France; 9 USDA/ARS Children’s Nutrition Research Center, Department of Pediatrics, Baylor College of Medicine, Houston, Texas, United States of America; 10 Cancer Epidemiology and Intelligence Division, Cancer Council Victoria, Melbourne, Australia; 11 Centre of Excellence in Cancer Prevention, School of Population & Public Health, Faculty of Medicine, The University of British Columbia, Vancouver, Canada; 12 Department of Mathematics and Statistics, Dordt College, Sioux Center, Iowa, United States of America; 13 Department of Biostatistics, University of Washington School of Public Health, Seattle, Washington, United States of America; 14 Department of Health Sciences, Faculty of Earth & Life Sciences, Vrije Universiteit Amsterdam, Amsterdam Public Health Research Institute, Amsterdam, the Netherlands; 15 Department of Medicine, Division of Cardiovascular Medicine, Stanford University School of Medicine, Stanford, California, United States of America; 16 Divisions of Aging, Department of Medicine, Brigham and Women’s Hospital and Harvard Medical School, Boston, Massachusetts, United States of America; 17 Division of Human Nutrition, Wageningen University, Wageningen, the Netherlands; 18 Centre for Epidemiology and Biostatistics, The University of Melbourne, Parkville, Australia; 19 Icelandic Heart Association Research Institute, Holtasmári 1, Kópavogur, Iceland, Iceland; 20 Department of Internal Medicine, Sanford School of Medicine, University of South Dakota, Sioux Falls, South Dakota, United States of America; 21 OmegaQuant Analytics LLC, Sioux Falls, South Dakota, United States of America; 22 National Institute for Health Research Biomedical Research Centres Core Nutritional Biomarker Laboratory, University of Cambridge, Addenbrooke’s Hospital, Cambridge, United Kingdom; 23 National Institute for Health Research Biomedical Research Centres Core Metabolomics and Lipidomics Laboratory, University of Cambridge, Addenbrooke’s Hospital, Cambridge, United Kingdom; 24 Medical Research Council Elsie Widdowson Laboratory, Cambridge, United Kingdom; 25 Institute of Clinical Medicine, Internal Medicine, University of Eastern Finland, Kuopio, Finland; 26 Department of Medicine, Kuopio University Hospital, Kuopio, Finland; 27 Department of Medical Sciences, Uppsala University, Uppsala, Sweden; 28 Department of Internal Medicine, National Taiwan University Hospital, Zhongzheng District, Taipei City, Taiwan; 29 Departments of Epidemiology and Medicine at the University of Iowa College of Public Health, Iowa City, Iowa, United States of America; 30 The New York Academy of Medicine, New York, New York, United States of America; 31 Center of Research on Psychology in Somatic Diseases, Department of Medical and Clinical Psychology, Tilburg University, Tilburg, the Netherlands; 32 Department of Laboratory Medicine and Pathology, University of Minnesota, Minneapolis, Minnesota, United States of America; 33 Public Health Sciences, Wake Forest School of Medicine, Winston-Salem, North Carolina, United States of America; 34 The George Institute for Global Health and the Faculty of Medicine, University of New South Wales, Sydney, Australia; 35 Friedman School of Nutrition Science and Policy, Tufts University, Boston, Massachusetts, United States of America; University of Exeter, UNITED KINGDOM

## Abstract

**Background:**

We aimed to investigate prospective associations of circulating or adipose tissue odd-chain fatty acids 15:0 and 17:0 and *trans*-palmitoleic acid, *t*16:1n-7, as potential biomarkers of dairy fat intake, with incident type 2 diabetes (T2D).

**Methods and findings:**

Sixteen prospective cohorts from 12 countries (7 from the United States, 7 from Europe, 1 from Australia, 1 from Taiwan) performed new harmonised individual-level analysis for the prospective associations according to a standardised plan. In total, 63,682 participants with a broad range of baseline ages and BMIs and 15,180 incident cases of T2D over the average of 9 years of follow-up were evaluated. Study-specific results were pooled using inverse-variance–weighted meta-analysis. Prespecified interactions by age, sex, BMI, and race/ethnicity were explored in each cohort and were meta-analysed. Potential heterogeneity by cohort-specific characteristics (regions, lipid compartments used for fatty acid assays) was assessed with metaregression. After adjustment for potential confounders, including measures of adiposity (BMI, waist circumference) and lipogenesis (levels of palmitate, triglycerides), higher levels of 15:0, 17:0, and *t*16:1n-7 were associated with lower incidence of T2D. In the most adjusted model, the hazard ratio (95% CI) for incident T2D per cohort-specific 10th to 90th percentile range of 15:0 was 0.80 (0.73–0.87); of 17:0, 0.65 (0.59–0.72); of *t*16:1n7, 0.82 (0.70–0.96); and of their sum, 0.71 (0.63–0.79). In exploratory analyses, similar associations for 15:0, 17:0, and the sum of all three fatty acids were present in both genders but stronger in women than in men (*p*_*interaction*_ < 0.001). Whereas studying associations with biomarkers has several advantages, as limitations, the biomarkers do not distinguish between different food sources of dairy fat (e.g., cheese, yogurt, milk), and residual confounding by unmeasured or imprecisely measured confounders may exist.

**Conclusions:**

In a large meta-analysis that pooled the findings from 16 prospective cohort studies, higher levels of 15:0, 17:0, and *t*16:1n-7 were associated with a lower risk of T2D.

## Abstract 2

Fumiaki Imamura and colleagues reveal a correlation between higher levels of circulating and adipose odd-chain fatty and trans-palmitoleic acids and lower risk of developing diabetes.

## Author summary

**Why was this study done?:**

- Effects of dairy fat on type 2 diabetes (T2D) are not well established. While dairy fat contains palmitic acid that could increase risk of T2D, it also contains several other types of fatty acids and further reflects specific foods, such as cheese or yogurt, that could reduce risk.
- Most prior studies of dairy foods and T2D have relied on self-reported dietary questionnaires, which may have errors or bias in memory as well as challenges in assessing less apparent sources of dairy fat such as in creams, sauces, cheeses, and cooking fats in mixed meals and prepared foods.
- Circulating and tissue biomarker concentrations of odd-chain saturated fats (15:0, 17:0) and natural ruminant *trans*-fats (*trans*-16:1n7) at least partly reflect dairy fat consumption, help capture multiple dietary sources without relying on memory or subjective reporting, and reflect a complementary approach to investigate associations with T2D.
- A consortium strategy combining all available studies maximises statistical power and generalizability, allows standardised analytical approaches and methods including of key population subgroups, and minimises potential for publication bias.

**What did the researchers do and find?:**

- We conducted a consortium project to pool new participant-level analyses of 16 cohort studies as part of the Fatty Acids and Outcomes Research Consortium (FORCE), including a total of 63,682 adults free of T2D at baseline, among whom 15,158 developed incident T2D over up to 20 years of follow-up.
- Participating studies conducted standardised analysis of the prospective associations between fatty acid biomarkers (15:0, 17:0, *trans*-16:1n7, and their sum) and the risk of developing T2D.
- Pooling all studies, each of the biomarkers and their sum were associated with lower risk of developing T2D, independently of major risk factors for T2D, including age, sex, race/ethnicity, socioeconomic status, physical activity, and obesity.
- For example, for the sum of these biomarkers, participants with higher levels experienced 29% (95% CI 21% to 37%) lower risk of T2D than adults with lower levels, comparing between the midpoints of the top fifth and the bottom fifth of concentrations.

**What do these findings mean?:**

- Higher circulating and tissue concentrations of odd-chain saturated fats and a natural ruminant *trans*-fat are associated with lower risk of T2D.
- While these biomarkers are known to reflect dairy fat consumption, their levels could also be influenced by other unknown factors. The findings support the need for investigation of determinants of levels of these fatty acids as well as health effects of dairy fat in interventional studies.
- Despite the several advantages of evaluating fatty acid biomarkers, the results cannot distinguish between different types of dairy foods (e.g., milk, cheese, yogurt, others), which could have differential effects.
- The findings provide the strongest evidence to date for relationships of these fatty acid biomarkers with T2D, informing the potential health effects and corresponding dietary recommendations for consumption of selected dairy products.

## Introduction

Regular consumption of dairy products is widely recommended in national and international guidelines as a major source of calcium and other minerals and vitamins as well as in low-income countries as a source of calories and protein. At least in high-income nations, fat-reduced dairy products are further recommended, rather than whole-fat products, with the aim of limiting calories and saturated fat [1]. However, these latter recommendations are primarily based on nutrient profiles of low-fat and whole-fat dairy products rather than empirical evidence on clinical effects of dairy fat from prospective observational studies or trials [2–8]. In clinical trials, consuming low-fat or free-fat dairy products does not consistently improve intermediate risk factors compared to consuming whole-fat or overall dairy products [2–4]. In observational studies, total dairy consumption has not been associated with cardiovascular diseases, without consistent distinction based on dairy fat content. Regardless of fat content, total dairy consumption has been associated with lower incidence of type 2 diabetes (T2D) [8], whereas evidence is inconsistent for different types of dairy foods such as milk, yogurt, and cheese.

Studies assessing dairy consumption using self-reported dietary questionnaires may be partly limited by misclassification or bias in reporting [9]. In addition, the common use of dairy products such as butter, milk, cheese, and cream in cooking, in mixed dishes (e.g., pizza), and bakery products (e.g., cakes) may substantially impede an accurate assessment of exposure to dairy fat. To reduce these limitations, measured biomarkers correlated with dairy fat consumption can be used, including circulating and adipose proportions of pentadecanoic acid (15-carbon saturated fatty acid, 15:0), heptadecanoic acid (17:0), and *trans*-palmitoleic acid (*t*16:1n7) [10–20]. Levels of these biomarkers correlate with self-reported consumption of total dairy, high-fat dairy, and dairy fat (*r* = 0.4 to 0.7) based on 24-hour recalls or 7-day food records [16–18]; are significantly increased in response to dairy consumption or decreased in replacing high-fat dairy with low-fat dairy in trials [19,20]; and are correlated with each other even though they represent two distinct fatty acid classes (the odd-chain saturated fats 15:0 and 17:0; the natural ruminant *trans*-fat *t*16:1n7) with divergent chemical structures and metabolism.

To date, several individual cohorts have published on associations of the odd-chain saturated fatty acids only [21] or odd-chain fatty acids and *t*16:1n7 together [13,14,22] with incidence of T2D. However, potential for publication bias cannot be excluded; individual studies may be underpowered to detect potential differences in associations by sex or other characteristics [8]. To address these limitations and provide new evidence on relationships between these biomarkers and T2D, we conducted a pooling project to test the hypothesis that higher concentrations of 15:0, 17:0, and *t*16:1n7 would be associated with lower incident T2D, evaluating adults free from T2D in prospective cohorts participating in the Fatty Acids and Outcomes Research Consortium (FORCE).

## Methods

### Cohorts and study variables

FORCE was formed within the framework of the Cohorts for Heart and Aging Research in Genomic Epidemiology consortium fatty acid working group to focus on relationships between fatty acid biomarkers and health outcomes (http://force.nutrition.tufts.edu/about) [23,24]. FORCE cohorts were identified through expert contacts with existing large cohorts and publications, with updating over time when new cohort publications were identified. For the current investigation, we included 16 prospective studies (cohorts, nested case-control studies, or nested case-cohort studies) that met the following inclusion criteria and agreed to participate: adult aged 18 years or older free from diabetes at the time of fatty acid assessment; circulating or adipose 15:0, 17:0, or *t*16:1n7; and follow-up for incident T2D (S1 Text). Other cohorts participating in FORCE [23,24] did not contribute to this study because data on these fatty acids and/or incident T2D were not available. All cohorts obtained institutional review board approval and informed consents from participants. Authors FI and AF have full access to the data that are available upon request to the central committee of FORCE.

A standardised analysis protocol (S2 Text) was developed and was provided to each participating cohort. It included inclusion criteria (adults aged 18 years or older, not with diabetes, and with data on fatty acids and incident T2D), exposures, covariates, effect modifiers, outcomes, and longitudinal analyses. Following this harmonised protocol, each cohort performed new analysis of individual-level data. Study-specific results were entered to a standardised electronic form and compiled centrally; the results were then pooled in meta-analysis [25].

Details of participating cohorts, study participants, fatty acid assessment, ascertainment of incident T2D, and relevant citations are presented in S1 Text; fatty acid concentrations were assessed with gas chromatography in each cohort in one or more lipid compartments, including erythrocyte phospholipids, plasma phospholipids, plasma cholesteryl esters, plasma triglycerides, total plasma, or adipose tissue. Fatty acid concentrations in each cohort were expressed as a percent of total fatty acids in each lipid fraction. In extended analysis of prior work [22], within-person correlations of phospholipid fatty acids were moderate over 6 and 13 years (*n* = 607) (*r* = 0.64 and 0.46 for 15:0, respectively; 0.66 and 0.47 for 17:0; and 0.59 and 0.45 for *t*16:1n7), consistent with other biometric risk factors such as blood pressure [26].

In most cohorts, incident T2D was ascertained based on one or more criteria (S1 Text), including fasting glucose ≥126 mg/dL (7.0 mmol/L); 2-hour post oral glucose tolerance test glucose ≥200 mg/dL (11.1 mmol/L); new use of insulin or oral hypoglycaemic medication assessed by participant reports, medication inventories, or registries (S1 Text); and fasting or nonfasting HbA1C concentration ≥6.5%. In the Melbourne Collaborative Cohort Study (MCCS) [27] and the Alpha Omega Cohort (AOC) [28], incident T2D was defined by self-reported physician diagnosis, use of antidiabetic medication, or both. InterAct defined incident T2D by adjudicating self-reported diagnosis of T2D or data linkage to disease registry [21]. In studies with time-to-event data, follow-up time was calculated from baseline (time of fatty acid measurement) to date of development of incident T2D, death from any cause, loss to follow-up, or censoring at end of follow-up—whichever came first.

### Statistical analysis in individual studies

Statistical analyses were prespecified to describe population characteristics and conduct prospective analyses of associations of the fatty acid biomarkers and incident T2D. The primary exposure variables were 15:0, 17:0, *t*16:1n7, and their sum (or if only two were available, the sum of the two). The sum was considered a biomarker of dairy fat intake, given the available evidence that these each of these fatty acids at least partly reflects dairy fat intakes [11–20] and that these fatty acids are mutually intercorrelated [12–16,22]. Pearson correlation coefficients were calculated between these fatty acids in each study and between fractions in different lipid compartments when available in the same cohort.

For prospective associations, Cox proportional hazard regression models were fitted to data from cohort or nested case-cohort studies. In the MCCS [27] without detailed time-to-event data for participants, logistic regression was used. The fatty acids were evaluated as a continuous linear variable in units of the study-specific 10th to 90th percentile range and, in a separate model, as a dummy categorical variable (quintile categories).

Covariates in all multivariable-adjusted analyses were prespecified. The primary model included age, sex, field site, race, education, occupation, physical activity, smoking, alcohol use, prevalent hypertension (treated or self-reported), prevalent dyslipidaemia (treated or self-reported), prevalent coronary heart disease, and self-reported health status. We obtained measures of association from two additional models: one further adjusting for adiposity measures (BMI and waist circumference) and the other further adjusting for circulating concentrations of triglycerides and palmitate (16:0), markers of hepatic de novo lipogenesis. Study-specific approaches were allowed for modelling some covariates (e.g., numbers of education categories, imputation for missing covariates), depending on availability and prior established cohort-specific approaches, to minimise confounding bias within each cohort [25]. Using the multivariable-adjusted model including adiposity measures, we obtained study-specific measures of effect modification by age, sex, BMI, and race/ethnicity (indicator categories with white race as the reference group) by evaluating the coefficient of a crossproduct term between each fatty acid variable and each of the prespecified factors.

### Meta-analysis

Study-specific regression coefficients and measures of precision (standard errors) from each of continuous and categorical terms were pooled with an inverse-variance–weighted meta-analysis to estimate summary relative risks (RRs) per the 10th to 90th percentile range and quintile categories. Between-study heterogeneity was expressed as I-squared [29]. Odds ratios estimated in a study without information on time to event were considered to approximate RRs, and RRs were assumed to represent hazard ratios as well. Four cohorts assessed fatty acids in two lipid compartments: the Prospective Investigation of the Vasculature in Uppsala Seniors (PIVUS) and the AOC evaluated plasma cholesteryl esters and plasma phospholipid fatty acids, and the Nurses’ Health Study (NHS) and the Health Professionals’ Follow-up Study (HPFS) evaluated total plasma and red blood cell phospholipid fatty acids. In the primary meta-analysis, not to double-count estimates from these cohorts, we used estimates of phospholipid fatty acids that were likely to reflect a longer-term exposure than the other compartments [30]. Estimates from separate fractions were obtained separately as stratified analysis by lipid fractions.

Cohort-specific coefficients of crossproduct terms were pooled by inverse-variance–weighted meta-analysis to test potential interactions. Because analyses of potential interactions by age, sex, race/ethnicity, or BMI were exploratory, we corrected for multiple testing with two-tailed alpha = 0.0031 (0.05; 4 fatty acid variables; 4 potential effect modifiers). Because interactions by sex were significant, we post hoc estimated sex-specific RRs by obtaining relevant statistics from each cohort. We also conducted metaregression and stratified meta-analyses to examine whether associations varied by study-specific characteristics, including lipid compartment, region (the United States, Europe/Australia, Asia), mean prevalence of dyslipidaemia, and numbers of fatty acids assessed. Meta-analyses were performed using Stata software version 14.2 (Stata Corp., College Station, Texas) with alpha = 0.05 unless otherwise specified.

## Results

The 16 prospective studies (7 in the US, 7 in Europe, 1 in Australia, 1 in Taiwan) included 63,682 participants without known diabetes at baseline, among whom 15,180 incident T2D cases were identified during an average 9 years of follow-up (Table 1). All studies followed middle-aged or older adults with baseline mean age in each cohort ranging from 49 to 76 years. Average BMIs ranged from 25.0 to 28.4 kg/m^2^ except for Taiwan with an average BMI of 23.3 kg/m^2^. Most studies included predominantly white participants, although meaningful numbers of nonwhites were included in the Cardiovascular Health Study (CHS; 11.0% nonwhite), the Multi-Ethnic Study of Atherosclerosis (MESA; 71.6% nonwhite), the Women’s Health Initiative Memory Study (WHIMS; 11.6% nonwhite), and the Taiwanese study (100% Asian).

**Table 1:** Baseline characteristics of 16 studies of the pooling analysis of fatty acid biomarkers (15:0, 17:0, *trans*-16:1n7) and incident T2D^a^.

Study	Country	Study design	Baseline year	Follow-up years, median	*N* adults (*N* cases)	Age, mean years	Sex, % women	BMI, mean (kg/m^2^)	Biomarker compartment	*N* fatty acids assessed
CHS	United States	Cohort	1992	10.6	3,179 (284)	75.1	61.5	26.4	PL	45
MESA	United States	Cohort	2000–2002	9.3	2,252 (309)	61.0	53.9	27.6	PL	27
IRAS	United States	Cohort	1992–1997	5.3	719 (146)	55.1	55.8	28.4	Total plasma	34
FHS	United States	Cohort	2005–2008	5.8	2,209 (98)	64.4	57.2	27.8	RBC PL	33
WHIMS	United States	Cohort	1996	11.0	6,510 (502)	70.1	100	28.1	RBC PL	22
NHS	United States	Cohort	1990	16.9	1,760 (177)	60.4	100	25.3	RBC PL, total plasma	37
HPFS	United States	Cohort	1994	11.1	1,519 (112)	64.1	0	25.8	RBC PL, total plasma	37
InterAct^b^	Eight European countries	Case cohort	1993–1997	12.3	27,296 (12,132)	52.3	62.3	26.0	PL	37
AGESR	Iceland	Cohort	2002–2006	5.2	753 (28)	75.5	59.5	27.0	PL	41
Three C	France	Cohort	1999–2000	8.0	565 (39)	76.0	64.3	25.0	RBC PL	35
AOC	The Netherlands	Cohort	2002–2006	2.5	760 (37)^c^	68.9	20.4	27.4	RBC PL, CE	38
ULSAM	Sweden	Cohort	1970–1973	21.4	2,009 (396)	54.4	0	25.2	Adipose tissue	17
PIVUS	Sweden	Cohort	2001–2004	10.0	879 (67)	72.5	51.0	26.7	PL, CE	16
METSIM	Finland	Cohort	2006–2010	5.5	1,302 (71)	57.3	0	26.4	PL	22
MCCS	Australia	Case cohort	1990–1994	4.0	6,151 (490)	56.3	53.9	27.0	PL	53
CCCC	Taiwan	Cohort	1992–1993	6.0	1,838 (128)	58.7	40.0	23.2	Total plasma	29

^a^Characteristics at the time of fatty acid biomarker measurement.

^b^Upon a decision within the cohort, InterAct provided pooled estimates from eight European countries: France, Spain, the United Kingdom, Sweden, Germany, Italy, Denmark, and the Netherlands.

^c^The AOC evaluated 1,741 participants (201 incident cases) with CE measures that were analysed in secondary analyses.

**Abbreviations**: AGESR, Age, Genes, Environment Susceptibility Study (Reykjavik); AOC, Alpha Omega Cohort; CCCC, Chin-Shan Community Cardiovascular Cohort Study; CE, cholesteryl ester; CHS, Cardiovascular Health Study; FHS, Framingham Heart Study; HPFS, Health Professionals’ Follow-up Study; MCCS, Melbourne Collaborative Cohort Study; MESA, Multi-Ethnic Study of Atherosclerosis; METSIM, Metabolic Syndrome in Men Study; NHS, Nurses’ Health Study; PIVUS, Prospective Investigation of the Vasculature in Uppsala Seniors; PL, phospholipid; RBC, red blood cell; Three C, Three City Study; ULSAM, Uppsala Longitudinal Study of Adult Men; WHIMS, Women’s Health Initiative Memory Study.

**Fig 1:**
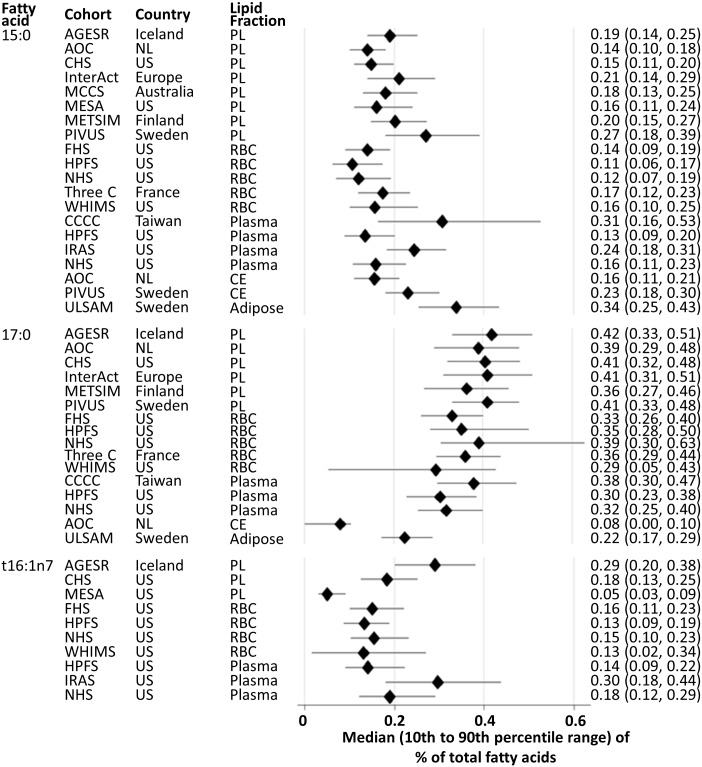
Proportions of fatty acid biomarkers for dairy fat consumption. Plots represent median (diamond) and ranges of the 10th to 90th percentiles (horizontal bar). See Table 1 for the abbreviations of cohorts. CE, cholesteryl ester; NL, the Netherlands; PL, phospholipid; RBC, red blood cell; t16:1n7, *trans*-16:1 n-7; US, United States.

Relative concentrations of 15:0, 17:0, and *t*16:1n7 were generally low (0.1% to 0.5 mol% of total fatty acids), as previously described (Fig 1) [13,14,21,22]. Correlations between 15:0, 17:0, and *t*16:1n7 ranged from 0.3 to 0.8, with the exception of *r* = 0.0 in the Insulin Resistance Atherosclerosis Study and WHIMS (S1 Table). Correlations of each of the fatty acids between two lipid fractions (e.g., phospholipids and total plasma; phospholipids and cholesteryl esters) were also moderate to strong (*r* = 0.39 to 0.75) (S2 Table).

**Fig 2:**
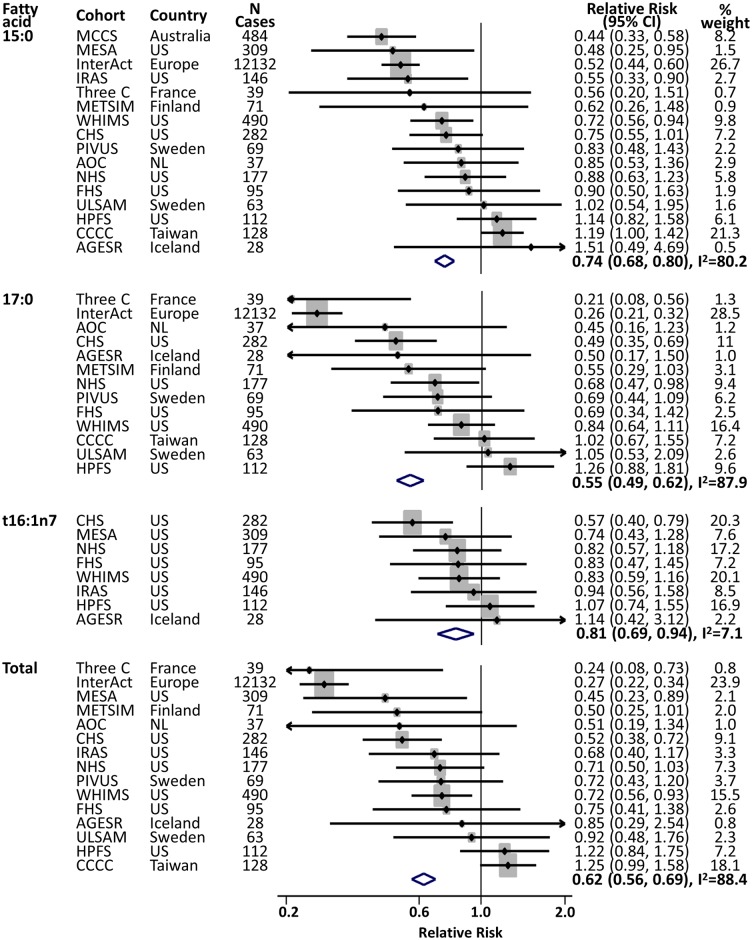
Prospective associations of fatty acid biomarkers for dairy fat consumption with the risk of developing T2D. RR and 95% CIs per cohort-specific range from the 10th to 90th percentiles are presented: dots from individual studies and diamonds as summary estimates pooled by inverse-variance–weighted meta-analysis. The sizes of the grey shaded areas represent relative contributions of each cohort to that summary estimate. Cohort-specific association was assessed in multivariable models in each cohort adjusting for sex, age, field site (if appropriate), race, socioeconomic status (education, occupation), smoking status, physical activity, alcohol consumption, family history of diabetes, dyslipidaemia, hypertension, menopausal status (only for women), prevalent coronary heart disease, BMI, and waist circumference. Models without the adiposity measures and models including palmitate (16:0) and triglycerides did not alter the results materially (S1 Fig). See Table 1 for the abbreviations of cohorts. NL, the Netherlands; RR, relative risk; T2D, type 2 diabetes mellitus; US, United States.

In meta-analysis of 15:0 (16 cohorts, 59,701 participants, 14,658 cases), higher 15:0 levels were associated with 26% lower risk of T2D (per 10th to 90th percentile range, pooled RR = 0.74 [95% CI 0.68–0.80]) adjusted for demographic, clinical, socioeconomic, and lifestyle variables (S1 Fig); 20% lower risk (RR = 0.80 [95% CI 0.74–0.87]) when further adjusted for adiposity measures (Fig 2); and 20% lower risk (RR = 0.80 [95% CI 0.73–0.87]) when further adjusted for biomarker concentrations of palmitic acid and triglycerides (S2 Fig). Inverse associations were also observed for 17:0 (13 cohorts, 50,579 participants, 13,720 cases), *t*16:1n7 (8 studies, 18,901 participants, 1,636 cases), and the sum of dairy biomarker fatty acids (15 studies, 53,550 participants, 14,175 cases). In post hoc sensitivity analyses excluding the study with the largest weight (InterAct or WHIMS, Fig 2), results were not substantially altered: RR per 10th to 90th percentile range (95% CI) for 15:0, 0.75 (95% CI 0.62–0.92); for 17:0, 0.73 (95% CI 0.55–0.96); for t16:1n7, 0.84 (95% CI 0.72–0.98); and for their sum, 0.75 (95% CI 0.57–0.99).

**Fig 3:**
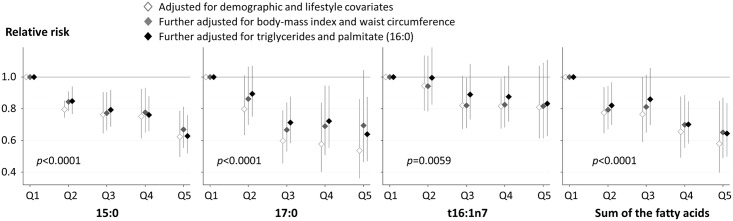
Prospective associations of quintile categories of fatty acid biomarkers for dairy fat consumption with the risk of developing T2D. Cohort-specific associations by quintiles were assessed in multivariable models in each cohort and pooled with inverse-variance–weighted meta-analysis. Cohort-specific multivariable adjustment was made. In the first model (open diamond), estimates were adjusted for sex, age, smoking status, alcohol consumption, socioeconomic status, physical activity, dyslipidaemia, hypertension, and menopausal status (only for women). Then, the estimates were further adjusted for BMI (grey diamond) and further adjusted for triglycerides and palmitic acid (16:0) as markers of de novo lipogenesis (black diamond). To compute *p*-values for a trend across quintiles, each fatty acid was evaluated as an ordinal variable in the most adjusted model. T2D, type 2 diabetes mellitus.

Results were similar evaluating risk across quintile groups of each fatty acid including in each multivariable model (Fig 3). Comparing the top to the bottom quintile of fatty acid levels in the fully adjusted model, RRs (95% CI) were 0.63 (0.52–0.76) for 15:0, 0.64 (0.47–0.87) for 17:0, 0.83 (0.62–1.11) for *t*16:1n7, and 0.65 (0.51–0.83) for their sum. Moderate to high heterogeneity was evident (I^2^ ranging from 60% to 90%) (Fig 2, S1 Fig, S3 Fig), except for *t*16:1n7 (I^2^ 0% to 7.7%). Results of post hoc analysis estimating random effects were similar (S3 Table).

In exploratory analyses, the inverse association with T2D was stronger in women than in men for 15:0 (*p*_*interaction*_ = 0.0003), 17:0 (*p*_*interaction*_ = 0.003), and the sum of the fatty acids (*p*_*interaction*_ = 0.0003), with women experiencing a 20% to 27% lower risk than men (S3 Table). For example, RR (95% CI) per 10th to 90th percentile range for 15:0 was 0.76 (0.69–0.84) for women and 0.93 (0.85–1.01) for men. Metaregression did not identify any other significant sources of heterogeneity (*p*_*interaction*_ > 0.1 each), including by geographic region, measured lipid compartment, prevalence of dyslipidaemia, or the number of fatty acids assessed (S3 Table).

## Discussion

This harmonised pooling project of participant-level data among 16 prospective cohort studies provides, to our knowledge, the most comprehensive evidence for associations of biomarker levels of 15:0, 17:0, and *t*16:1n7 with risk of T2D. Comparing the top to the bottom quintile of participants in each cohort, we found that higher levels of the sum of these fatty acids were associated with approximately 30% lower risk of developing T2D. This relationship remained significant after adjustment for demographic characteristics, socioeconomic status, lifestyle factors, medical history, adiposity measures, and biomarkers of de novo lipogenesis.

Measured circulating and tissue levels of 15:0, 17:0, and *t*16:1n7 are free from bias in relation to memory or reporting. Compared with estimated dairy fat intake from self-reported questionnaires, direct measurement also facilitates assessment of exposure to numerous ‘hidden’ sources of dairy fat in the food supply, e.g., as found in many dishes that include varying amounts of sauces, creams, and butter, milk, or cheese as mixed or prepared meals. Odd-chain saturated fats can be found in other foods, such as meat or fish [31,32], and their blood levels are measurable among self-reported vegans [10]. However, levels among vegans are significantly lower than among lacto-ovo vegetarians, supporting a sensitivity of the biomarkers to dairy fat consumption [10]. Several additional lines of evidence support a role of these fatty acids as biomarkers reflecting consumption of dairy fat and high-fat dairy products. First, among different food groups, correlations of these fatty acid biomarkers are strongest with dairy foods and dairy fat [12,15,16]. Such correlations are generally low to modest (*r* = 0.1 to 0.5) in studies using food-frequency questionnaires (which may miss many ‘hidden’ sources of dairy fat) [12,13,21] but much stronger (*r* = 0.4 to 0.7) in studies evaluating 24-hour recalls or 7-day food records, which much more completely capture the types and details of specific foods consumed [16–18]. Second, in controlled interventions, levels of these fatty acids are significantly increased or decreased in response to even moderate changes in dairy fat consumption [11,19]. Third, these two very different classes of fatty acids—the odd-chain saturated fats 15:0 and 17:0, and the natural ruminant *trans*-fat *t*16:1n7—are intercorrelated with each other and also similarly associated with T2D. If either endogenous metabolic influences or non-dairy dietary sources were a primary determinant of their levels, little plausible rationale would exist for a meaningful interrelation of these biochemically and metabolically unrelated fatty acids. Finally, while as a biomarker of dairy fat the circulating levels of these fatty acids could also be influenced by meat or fish consumption [10], such foods are not associated with lower risk of T2D in Western populations (and red meat is associated with higher risk) [33,34], so that such influences would weaken associations of these fatty acids with T2D.

A small crossover trial (*n* = 16) recently evaluated potential endogenous production of 15:0 and 17:0 from dietary fibre (inulin) and propionate (a short-chain [3-carbon] fatty acid) in comparison to cellulose [35,36]. The primary randomised comparison did not identify any significant effect of these factors on 15:0 or 17:0 levels. In secondary analyses evaluating pre-post (nonrandomised) levels, inulin intake was associated with higher levels of 17:0, while propionate was associated with higher 15:0 and 17:0; this was further supported by an accompanying in vitro–controlled experiment suggesting elongation of propionate into 15:0 and 17:0 using liver cancer cells [35]. The major dietary source of propionate is cheese (157 mg per 100 g), in particular Swiss cheese (311 mg per 100 g), with far lower levels in other dairy foods such as milk, yogurt, and cream (2–9 mg per 100 g) and even lower levels in other major food groups (<1 mg per 100 g) (S4 Table) [37]. High levels in cheese are plausibly related to the presence of propionate-producing bacteria and the use of sodium propionate and other propionate salts as natural preservatives and mould inhibitors in cheese [37]. These findings further support the role of odd-chain saturated fatty acids as biomarkers of dairy fat consumption, both as contained in dairy fat and as potentially synthesised from propionate in cheese [35,36].

While 15:0 has a modestly stronger correlation with self-reported dairy foods than 17:0 in some prior studies [10,12,13,16–18], we found that 17:0 was more strongly associated with lower risk of T2D. Reasons for this are unclear but could reflect differences in blood lipid compartments assessed, 17:0 being a better measure of ‘hidden’ dairy fat in mixed foods, or possible differences in metabolic influences as mentioned above [35,38].

Our findings support the need for careful investigation to elucidate the potential biological mechanisms underlying the observed lower risk of T2D. Odd-chain fatty acids and *t*16:1n7 have structural similarity to 16:0 and may interfere with lipotoxic effects of 16:0 on the pancreas [39]; it has also been hypothesised that *t*16:1n7 may mimic *cis*-16:1n7 and suppress hepatic de novo lipogenesis [13]. These fatty acids may also be a marker for other beneficial compounds in dairy fat or dairy-fat–rich foods such as cheese [40]. Examples of relevant constituents could include magnesium, which appears to improve hyperglycaemia and insulin resistance [41], and oestrogens, which are naturally present in dairy products [42,43] and which may reduce the risk of T2D [43], as shown in two trials recruiting postmenopausal women [44,45]. However, these prior trials tested supradietary doses of magnesium (>250 mg/day) and oestrogens (3 mg/day) [41,44,45] compared with typical doses in dairy foods, approximately 20 mg and <0.01 mg, respectively, in 150 g of milk or yogurt, for example [42,43]. Probiotics such as in yogurt lower glucose and HbA1c in trials [40,46], suggesting relevant interactions between probiotics, short-chain fatty acids, gut microbiota, and T2D [35,40]. Fermented milk and cheeses are also linked to lower risk of T2D [47], suggesting potential metabolic benefits of vitamin K2 or other compounds produced during fermentation [40]. Other constituents of dairy hypothesised to improve metabolic risk include vitamin D and calcium, but for which supplement trials do not support antidiabetic effects [48], branched-chain amino acids, but for which limited evidence suggests potential harms on insulin sensitivity [49], and animal protein, but which is not associated with lower risk of T2D [50]. Given the prevalence of dairy foods in the food supply and the prevailing conventional wisdom to avoid dairy fat, our results indicate a clear need for further clinical and biochemical investigations on 15:0, 17:0, *t*16:1n7, and other components of dairy fats to clarify the mechanisms underlying our observations and help better understand roles of dairy consumption for the prevention of T2D and related diseases.

In exploratory analyses, the inverse association of 15:0 and 17:0 with T2D was stronger in women than in men. Consistent with this, a meta-analysis of self-reported consumption of dairy products suggested stronger protective associations of yogurt consumption with T2D risk in women than in men (RR per 50 g/day = 0.89 in women and 0.97 in men; *p*_heterogeneity_ = 0.03) [8]. If confirmed in future studies, such an interaction may help elucidate potential mechanisms of benefit, e.g., pathways related to sex steroids [51] or starch and sugar intake (as a substitute for dairy fat) [52] and corresponding effects on atherogenic dyslipidaemia, visceral adiposity, and insulin resistance [52].

Our analysis has several strengths. Use of biomarkers provided measures free of limitations in self-reported dietary exposure. The similar results from several fatty acids linked to dairy fat increased confidence in the specificity of our findings. Our collaborative pooling of cohorts across different continents led to large numbers of studies, participants, and events, increasing both generalisability and statistical power. The pooling of all available cohorts minimised potential for publication bias of just the few individually significant cohorts. The standardised definitions and modelling of the populations, exposures, outcomes, and multivariable-adjusted analyses minimised bias and heterogeneity due to methodological considerations.

Potential limitations deserve consideration. The timing of diagnosis of T2D can be delayed, causing misclassification of timing in survival analysis. However, most cohorts included regular study visits and many included regular glycaemic measurements, reducing such misclassification in comparison to clinical practice. Also, any delays in diagnosis would likely be random with respect to baseline measures of fatty acid biomarkers, causing bias toward the null and increased uncertainty in estimates. Fatty acid biomarkers were assessed at baseline in each cohort, and variability over time would lead to regression dilution bias of associations toward the null. The biomarkers, despite several advantages, cannot distinguish between different food sources of dairy fat (e.g., cheese, yogurt, milk) or other foods. As an alternative to pooling of standardised participant-level analysis, all individuals could have been combined into a single dataset. Such an analysis would have a larger statistical power than our two-stage approach but require stronger assumptions, such as about covariate effects being constant across all studies [25]. Unmeasured or imprecisely measured factors may cause residual confounding, although we adjusted for major potential confounders including obesity and triglyceride levels and confirmed little difference in results across different models. Additionally, while high consumption of dairy products may be correlated with health consciousness or healthy dietary patterns in some populations [53], health-conscious consumers may have been more likely to consume low-fat than whole-fat dairy during the time periods of these studies given the prevailing dietary recommendations. Therefore residual confounding, if present, may cause underestimation of the strength of the inverse associations. As in many meta-analyses, between-study heterogeneity was evidenced and could not be fully explained. The large numbers of cases in many cohorts increased the precision of each within-study estimate, which increases the chances of finding even unimportant heterogeneity. Heterogeneity could also partly relate to varying degrees of intercorrelations between fatty acids and between tissues as well as underlying differences in populations, dietary patterns, and varieties of dairy products, including processing and fat contents. We had more limited data in nonwhite populations, requiring further research in diverse populations for which different types of dairy products may be consumed with different preparation methods.

In summary, our consortium of 16 prospective cohort studies identified significant associations of higher concentrations of 15:0, 17:0, and *t*16:1n7 with lower incidence of T2D. These novel findings support the need for additional clinical and molecular research to elucidate the potential effects of these fatty acids on glucose–insulin metabolism and the potential role of selected dairy products for the prevention of T2D.

## Supporting information

S1 Table Correlations between fatty acid biomarkers for dairy fat consumption. (DOCX)

S2 Table Correlations between fatty acid biomarkers for dairy fat consumption of two lipid fractions. (DOCX)

S3 Table Prospective associations of fatty acid biomarkers for dairy fat consumption with the risk of developing T2D: Stratified analyses by regions, lipid fractions, prevalence of dyslipidaemia, and the number of fatty acids measured. (DOCX)

S4 Table Average amounts of naturally occurring propionate in selected foods. (DOCX)

S1 Fig Prospective associations of fatty acid biomarkers for dairy fat consumption with the risk of developing T2D. (TIF)

S2 Fig Prospective associations of fatty acid biomarkers for dairy fat consumption with the risk of developing T2D after adjustment for adiposity measures, palmitic acid, and triglycerides. (TIF)

S1 Text Characteristics of prospective cohorts evaluating associations of fatty acid biomarkers for dairy fat consumption with the risk of developing T2D. (DOCX)

S2 Text Study protocol. (DOCX)

S1 Checklist PRISMA checklist. (DOCX)

## Abbreviations

AGESR: Age, Genes, Environment Susceptibility Study (Reykjavik)
AOC: Alpha Omega Cohort
CCCC: Chin-Shan Community Cardiovascular Cohort Study
CHS: Cardiovascular Health Study
FHS: Framingham Heart Study
FORCE: Fatty Acids and Outcomes Research Consortium
HPFS: Health Professionals’ Follow-up Study
MCCS: Melbourne Collaborative Cohort Study
MESA: Multi-Ethnic Study of Atherosclerosis
METSIM: Metabolic Syndrome in Men Study
NHS: Nurses’ Health Study
PIVUS: Prospective Investigation of the Vasculature in Uppsala Seniors
RR: relative risk
T2D: type 2 diabetes mellitus
Three C: Three City Study
ULSAM: Uppsala Longitudinal Study of Adult Men
WHIMS: Women’s Health Initiative Memory Study
