# Supplementary material for: Fatty acid biomarkers of dairy fat consumption and incidence of type 2 diabetes: A pooled analysis of prospective cohort studies
Source: PLoS Med. 2018 Oct 10;15(10):e1002670. doi: 10.1371/journal.pmed.1002670 (PMC6179183; doi:10.1371/journal.pmed.1002670)
Supplement: S1 Table — (DOCX) [file pmed.1002670.s001.docx]

| **S1 Table. Correlations between** fatty acid biomarkers for dairy fat consumption | | | | | | | | |
| --- | --- | --- | --- | --- | --- | --- | --- | --- |
| **Study^1^** | **Fraction^2^** | **N** | **Pearson correlation coefficients between two fatty acid variables^3^** | | | | | |
|  |  |  | **15:0 and 17:0** | **15:0 and 16:1n7t** | **17:0 and 16:1n7t** | **15:0 and total** | **17:0 and total** | **16:1n7t and total** |
| AGESR | PL | 753 | 0.62 | 0.75 | 0.80 | 0.82 | 0.93 | 0.95 |
| CHS | PL | 3179 | 0.41 | 0.49 | 0.65 | 0.74 | 0.87 | 0.86 |
| InterAct | PL | 15919 | 0.55 |  |  | 0.83 | 0.93 |  |
| PIVUS | PL | 879 | 0.38 |  |  | 0.86 | 0.78 |  |
| MESA | PL | 2252 |  | 0.30 |  | 0.91 |  | 0.66 |
| METSIM | PL | 1302 | 0.53 |  |  | 0.83 | 0.92 |  |
| FHS | RBC PL | 2209 | 0.42 | 0.45 | 0.43 | 0.76 | 0.81 | 0.79 |
| Three C | RBC PL | 565 | 0.57 |  |  | 0.84 | 0.92 |  |
| NHS | RBC PL | 1760 | 0.54 | 0.58 | 0.69 | 0.75 | 0.92 | 0.84 |
| HPFS | RBC PL | 1519 | 0.69 | 0.67 | 0.72 | 0.84 | 0.94 | 0.86 |
| AOC | RBC PL | 795 | 0.48 |  |  | 0.77 | 0.93 |  |
| WHIMS | RBC PL | 5668 | -0.06 | -0.06 | 0.38 | 0.35 | 0.79 | 0.71 |
| NHS | Plasma | 1848 | 0.48 | 0.48 | 0.43 | 0.76 | 0.78 | 0.82 |
| HPFS | Plasma | 1471 | 0.66 | 0.26 | 0.52 | 0.83 | 0.86 | 0.81 |
| CCCC | Plasma | 1838 | 0.26 |  |  | 0.89 | 0.61 |  |
| IRAS | Plasma | 719 |  | -0.02 |  | 0.48 |  | 0.87 |
| ULSAM | Adipose | 2009 | 0.57 |  |  | 0.94 | 0.82 |  |
| AOC | CE | 4741 | 0.39 |  |  | 0.86 | 0.81 |  |
| Average^4^ |  |  | 0.45 | 0.34 | 0.54 | 0.80 | 0.88 | 0.90 |

^1^ AGES-Reykjavik, Age, Genes, Environment Susceptibility Study (Reykjavik); AOC, Alpha Omega Cohort; CCCC, Chin-Shan Community Cardiovascular Cohort Study; CHS, Cardiovascular Health Study; FHS, Framingham Heart Study; HPFS, Health Professionals’ Follow-up Study; MESA, Multi-Ethnic Study of Atherosclerosis; METSIM, Metabolic Syndrome in Men Study; NHS, Nurses’ Health Study; PIVUS, Prospective Investigation of the Vasculature in Uppsala Seniors; Three C, Three City Study; ULSAM, Uppsala Longitudinal Study of Adult Men; WHIMS, Women’s Health Initiative Memory Study. Melbourne Collaborative Cohort Study and cholesteryl esters components of PIVUS measured a single dairy fatty acid, and thus correlation coefficients were not available.
^2^ CE, cholesteryl esters; PL, phospholipids; RBC, red blood cells

^3^ Correlation coefficients between four variables of interest: 15:0, 17:0, trans 16:1n7 (16:1n7t), and sum of these three (or two if one was not assessed) (Total). Blank cells indicate no availability of one or two fatty acid variables for correlation coefficients.
^4^ Each average of correlation coefficients was calculated through Fisher Z-transformation.
