# Supplementary material for: Fatty acid biomarkers of dairy fat consumption and incidence of type 2 diabetes: A pooled analysis of prospective cohort studies
Source: PLoS Med. 2018 Oct 10;15(10):e1002670. doi: 10.1371/journal.pmed.1002670 (PMC6179183; doi:10.1371/journal.pmed.1002670)
Supplement: S2 Table — (DOCX) [file pmed.1002670.s002.docx]

| **S2 Table. Correlations between** fatty acid biomarkers for dairy fat consumption of **two lipid fractions** | | | | | |
| --- | --- | --- | --- | --- | --- |
| **Pair of fraction and study^1^** | **N** | **Correlations between two fatty acid fractions** | | | |
|  |  | **15:0** | **17:0** | **16:1n7t** | **Sum** |
| Erythrocyte phospholipids and total plasma | | | | |  |
| NHS | 1750 | 0.39 | 0.46 | 0.46 | 0.50 |
| HPFS | 1468 | 0.52 | 0.52 | 0.65 | 0.39 |
| Plasma phospholipids and cholesteryl esters | | | | |  |
| AOC | 795 | 0.75 | 0.49 | Not assessed | Not assessed |

^1^ AOC, Alpha Omega Cohort; HPFS, Health Professionals’ Follow-up Study; NHS, Nurses’ Health Study. Correlation coefficients in each of 15:0, 17:0, and trans 16:1n7 (16:1n7t) between two lipid fractions concentrations.
