# Supplementary material for: Fatty acid biomarkers of dairy fat consumption and incidence of type 2 diabetes: A pooled analysis of prospective cohort studies
Source: PLoS Med. 2018 Oct 10;15(10):e1002670. doi: 10.1371/journal.pmed.1002670 (PMC6179183; doi:10.1371/journal.pmed.1002670)
Supplement: S3 Table — (DOCX) [file pmed.1002670.s003.docx]

# **S3 Table. Prospective associations of** fatty acid biomarkers for dairy fat consumption **with the risk of developing type 2 diabetes: stratified analyses by regions, lipid fractions, prevalence of dyslipidemia, and the number of fatty acid measured.**

|  | **N studies^1^** | **Pentadecanoic acid**  **(15:0)** | | **Heptadecanoic acid**  **(17:0)** | | ***Trans*-palmitoleic acid**  **(*trans* 16:1n7)** | | **Sum of the fatty acid biomarkers** | |
| --- | --- | --- | --- | --- | --- | --- | --- | --- | --- |
|  |  | **RR (95% CI)**^2^ | ***p*^2^** | **RR (95% CI)** | ***p*** | **RR (95% CI)** | ***p*** | **RR (95% CI)** | ***p*** |
| Main pooled estimates | 16 | 0.80 (0.74, 0.87) |  | 0.61 (0.55, 0.68) |  | 0.82 (0.70, 0.95) |  | 0.67 (0.61, 0.74) |  |
| with a random-effect estimate^3^ | 16 | 0.80 (0.67, 0.95) |  | 0.71 (0.53, 0.95) |  | 0.82 (0.70, 0.95) |  | 0.71 (0.56, 0.89) |  |
| Regions |  |  |  |  |  |  |  |  |  |
| United States | 7 | 0.81 (0.70, 0.93) |  | 0.86 (0.73, 1.01) |  | 0.81 (0.69, 0.95) |  | 0.76 (0.66, 0.88) |  |
| Europe/Australia | 8 | 0.65 (0.58, 0.75) |  | 0.44 (0.37, 0.51) |  | 1.26 (0.44, 3.63) |  | 0.45 (0.39, 0.53) |  |
| Asia | 1 | 1.21 (1.01, 1.46) | 0.047 | 0.88 (0.57, 1.37) | 0.21 | Not available | 0.46 | 1.26 (0.98, 1.61) | 0.10 |
| Lipid fractions^4^ |  |  |  |  |  |  |  |  |  |
| Phospholipids | 13 | 0.72 (0.65, 0.79) |  | 0.59 (0.53, 0.66) |  | 0.82 (0.70, 0.96) |  | 0.59 (0.53, 0.66) |  |
| Total plasma | 4 | 1.16 (1.00, 1.33) |  | 0.81 (0.64, 1.04) |  | 1.00 (0.78, 1.28) |  | 1.07 (0.90, 1.28) |  |
| Cholesteryl esters | 2 | 0.94 (0.74, 1.20) |  | 1.40 (0.97, 2.02) |  | Not available |  | 1.14 (0.79, 1.64) |  |
| Adipose tissue | 1 | 1.04 (0.54, 1.99) | 0.44 | 1.13 (0.57, 2.25) | 0.55 | Not available | 0.95 | 0.97 (0.50, 1.89) | 0.39 |
| Dyslipidemia prevalence |  |  |  |  |  |  |  |  |  |
| <15%^5^ | 10 | 0.91 (0.81, 1.02) |  | 0.88 (0.73, 1.06) |  | 0.89 (0.73, 1.09) |  | 0.94 (0.81, 1.09) |  |
| ≥15% | 6 | 0.71 (0.63, 0.80) | 0.73 | 0.51 (0.45, 0.58) | 0.42 | 0.73 (0.58, 0.92) | 0.47 | 0.53 (0.46, 0.60) | 0.61 |
| N fatty acids assessed |  |  |  |  |  |  |  |  |  |
| <34^5^ | 7 | 0.97 (0.85, 1.12) |  | 0.86 (0.71, 1.05) |  | 0.81 (0.61, 1.08) |  | 0.90 (0.77, 1.05) |  |
| ≥34 | 9 | 0.72 (0.64, 0.80) | 0.33 | 0.53 (0.46, 0.60) | 0.29 | 0.82 (0.69, 0.99) | 0.55 | 0.55 (0.49, 0.63) | 0.57 |
| Exclusion of the most weighted cohort (EPIC-InterAct) | 15 | 0.87 (0.79, 0.96) |  | 0.82 (0.72, 0.94) |  | 0.82 (0.70, 0.95) |  | 0.84 (0.75, 0.94) |  |
| Sex^6^ |  |  |  |  |  |  |  |  |  |
| Men | 13 | 0.93 (0.85, 1.01) |  | 0.60 (0.53, 0.68) |  | Not estimated^6^ |  | 0.77 (0.70, 0.86) |  |
| Women | 12 | 0.76 (0.69, 0.84) | 0.0003 | 0.57 (0.49, 0.66) | 0.003 |  |  | 0.67 (0.60, 0.76) | 0.0003 |

^1^ The numbers varied by fatty acid variable, and the total number of cohorts used is presented. The total number of cohorts was sixteen.

^2^ Relative risk (RR) and 95% confidence interval (CI) per 10^th^ to 90^th^ percentile range in each cohort are presented. P values for heterogeneity between strata were obtained using meta-regression. As exception, *p* for heterogeneity by sex was estimated for meta-analysis of cross-product terms of fatty acid × sex estimated from participating cohorts in nine cohorts recruiting both men and women (heterogeneity between the sex-specific estimates would not necessarily comparable because of involvement of estimates from both men-only cohorts and women-only cohorts^6^ ).^3^ Estimated with random-effect meta-analysis.^4^ In the analysis stratifying lipid fractions, cohorts providing estimates of multiple lipid fractions (phospholipids and total plasma [NHS and HPFS] or cholesteryl esters [AOC and PIVUS]) contributed to multiple strata. In the meta-regression to assess heterogeneity, only phospholipid estimates from those cohorts were used to avoid double counting. Meta-analyses double-counting estimates from single cohorts, being over-powered, still produced null findings.

^5^ Median values were selected.

^6^ Sex-specific estimates were obtained by using parameter estimates for variables of the fatty acid and the cross-product term, accounting for variance and covariance measures. Variance-covariance matrix was obtained only for the significant interaction (15:0, 17:0, and sum of the fatty acids), and therefore sex-specific estimates for *trans* 16:1n7 were not available. The number of studies for men was based on the number of studies of men only or men and women; and that for women, the number of studies of women only or men and women.
