# Supplementary material for: Fatty acid biomarkers of dairy fat consumption and incidence of type 2 diabetes: A pooled analysis of prospective cohort studies
Source: PLoS Med. 2018 Oct 10;15(10):e1002670. doi: 10.1371/journal.pmed.1002670 (PMC6179183; doi:10.1371/journal.pmed.1002670)
Supplement: S4 Table — (DOCX) [file pmed.1002670.s004.docx]

S4 Table. Average amounts of naturally occurring propionate in selected foods ^1^

| **Food groups and items** | **Serving** | **Propionate** |  | **Food groups and items** | **Serving** | **Propionate** |
| --- | --- | --- | --- | --- | --- | --- |
| Dairy products |  |  |  | Other products |  |  |
| Milk / milk products | 100 g | 9.0 mg |  | Berries | 100 g | 0.002 mg |
| Yoghurt | 100 g | 2.2 mg |  | Mushroom | 100 g | 0.44 mg |
| Cheese, general | 100 g | 156.5 mg |  | Fish | 100 g | 0.35 mg |
| Cheese, blue | 100 g | 28.7 mg |  | Fish sauce | 10 g | 0.23 mg |
| Cheese, cheddar | 100 g | 162.0 mg |  | Shellfish | 100 g | 9.7 mg |
| Cheese, | 100 g | 310.5 mg |  | Lamb and mutton | 100 g | <0.01 mg |
| Lamb and mutton | 100 g | <0.01 mg |  | Wine | 100 g | 1.0 mg |
|  |  |  |  | Coffee, beans | 100 g | 8.77 mg |
|  |  |  |  | Coffee, beverages | 100 g | 0.49 mg |
|  |  |  |  | Wine and apple vinegar | 10 g | 0.70 mg |
|  |  |  |  | Soy sauce | 10 g | 1.06 mg |

^1^ Adopted from European Food Safety Authority (EFSA), Scientific Opinion on the re-evaluation of propionic acid (E 280), sodium propionate (E 281), calcium propionate (E 282) and potassium propionate (E 283) as food additives, EFSA J, 2014;12(7):3779.
