# Supplementary figures and images for: Fatty acid biomarkers of dairy fat consumption and incidence of type 2 diabetes: A pooled analysis of prospective cohort studies
Source: PLoS Med. 2018 Oct 10;15(10):e1002670. doi: 10.1371/journal.pmed.1002670 (PMC6179183; doi:10.1371/journal.pmed.1002670)

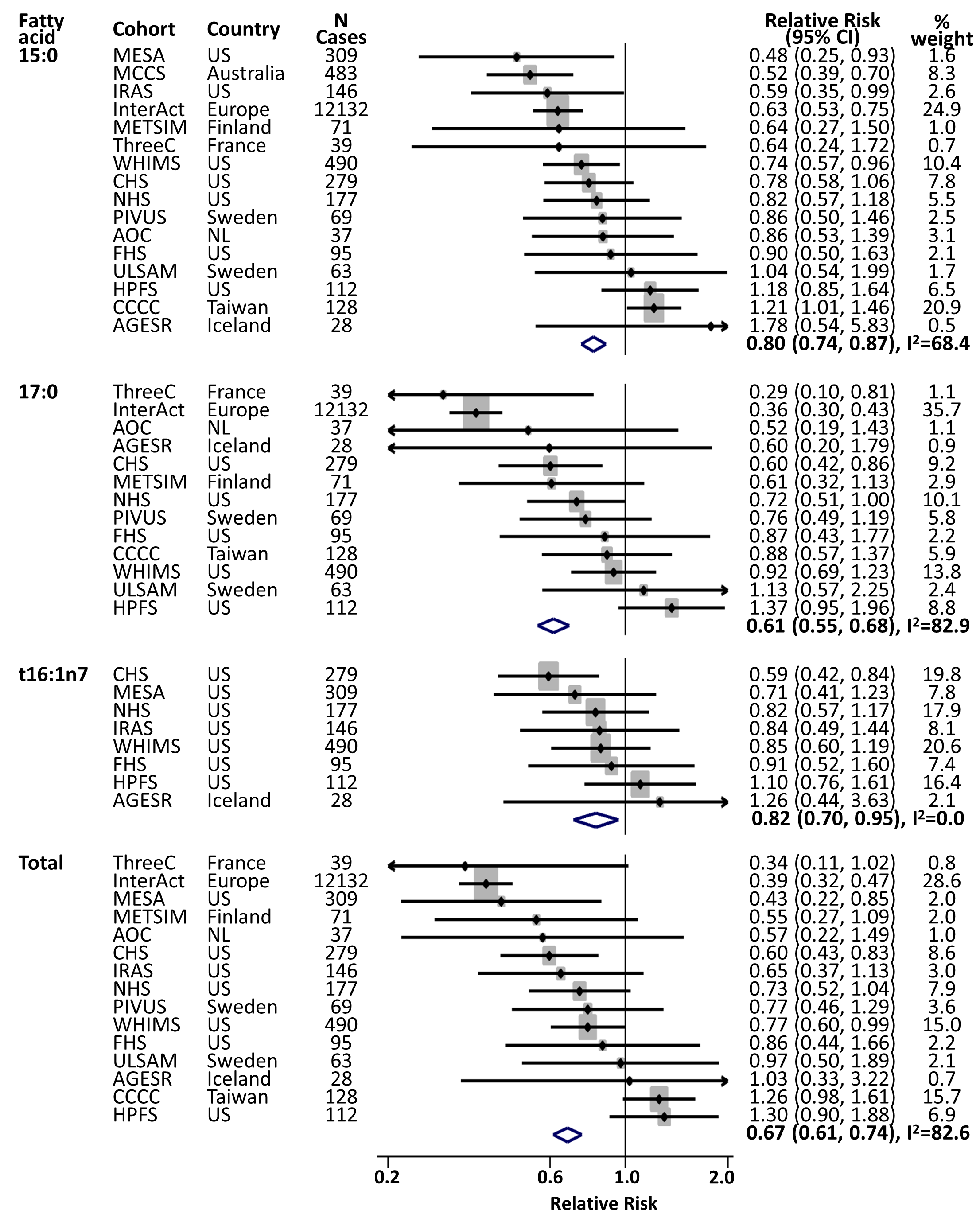

Supplement: S1 Fig — (TIF) [file pmed.1002670.s005.tif]

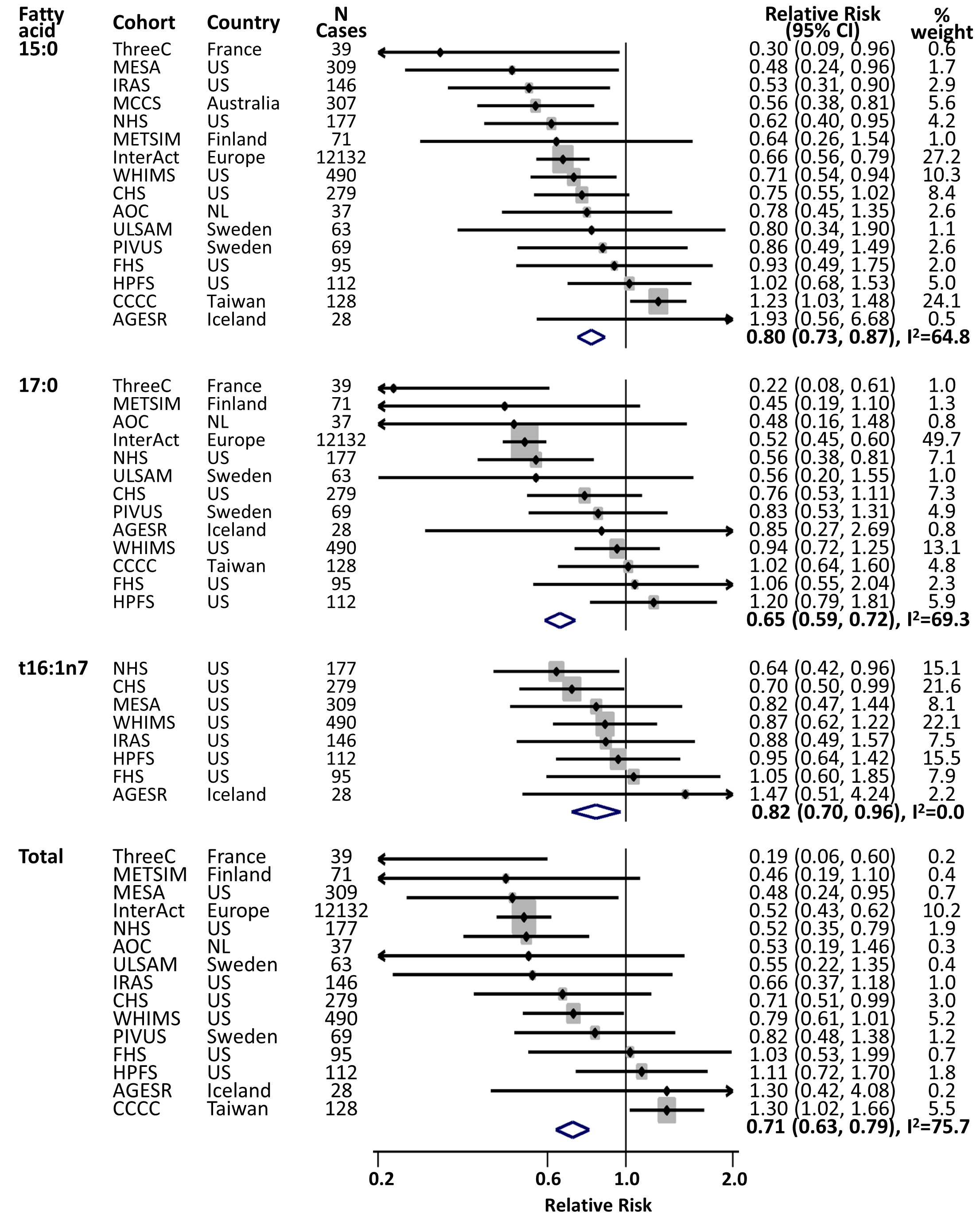

Supplement: S2 Fig — (TIF) [file pmed.1002670.s006.tif]
