# Supplementary material for: Fatty acid biomarkers of dairy fat consumption and incidence of type 2 diabetes: A pooled analysis of prospective cohort studies
Source: PLoS Med. 2018 Oct 10;15(10):e1002670. doi: 10.1371/journal.pmed.1002670 (PMC6179183; doi:10.1371/journal.pmed.1002670)
Supplement: S1 Text — (DOCX) [file pmed.1002670.s007.docx]

S1 Text. Characteristics of prospective cohorts evaluating associations of fatty acid biomarkers for dairy fat consumption with the risk of developing type 2 diabetes.
 **Age, Gene/Environment Susceptibility-Reykjavik study (AGESR), Ireland**^1,2^

*Cohort description*: The AGESR is a random sample of 5,764 men and women who were drawn from an established single center population based cohort; the Reykjavik Study, begun in 1967 to study heart disease. AGES-Reykjavik Study was designed to examine risk factors, including genetic susceptibility and gene/environment interaction, in relation to disease and disability in old age. At study baseline (2002–2006), participants were aged 66–96 years. A total of 753 adults with available data on circulating fatty acids and diabetes were eligible for the current analysis.

*Fatty acid measurement*: Blood samples were collected at baseline after an overnight fast and stored at -80 °C. Fatty acids were measured in plasma phospholipids at the Fred Hutchinson Cancer Research Center. Phospholipids were separated from other lipids by using one-dimensional thin-layer chromatography. Fatty acid methyl esters were prepared by direct transesterification and separated by using gas chromatography (Agilent Technologies 7890 Gas Chromatograph flame ionization detector detector; Supelco fusedsilica 100-m capillary column SP-2560; initially at 160 °C for 16 min, ramped up at 3.0 °C/min to 240 °C, and held for 15 min). The identification, precision, and accuracy were continuously evaluated by using both model mixtures of known fatty acid methyl esters and established in-house control pools. Fatty acids were expressed as the weight percentage of the total phospholipid fatty acids analyzed. The CV from pooled quality-control samples for major polyunsaturated fatty acids were all 2.5%. CVs for other major fatty acids were 0.77% (palmitic), 0.47% (stearic), and 0.42% (oleic).

*Outcome ascertainment*: Incident diabetes were determined from self-reported diabetes, diabetes medication use or fasting plasma glucose ≥7 mmol/L based on American Diabetes Association diagnosis recommendations.

**Alpha Omega Cohort (AOC), the Netherlands**^3,4^

*Cohort description*: AOC is a cohort of 4837 non-hospitalized patients who experienced a myocardial infarction up to 10 years before enrolment. The study includes a trial phase (Alpha Omega Trial, 3-year intervention with low doses of n-3 fatty acids, until 2009). It is now used as prospective cohort study for risk prediction in post-MI patients. The patients were recruited in collaboration with cardiologists from 32 Dutch hospitals. At baseline (2002-2006), data were collected on diet, lifestyle, cardiovascular risk factors, medical history, and medication use. Subjects were physically examined by trained research nurses, which included anthropometry, blood pressure, heart rate, and blood sampling. Examinations were repeated after 20 months (midterm examination in n=800) and 40 months (final examination). Patients have been continuously followed for cause-specific mortality, also after the trial ended.

*Fatty acid measurement*: Baseline blood samples of 10 mL of non-fasting venous blood were drawn at the patients’ home or at the hospital. For cholesteryl ester fatty acid analysis, blood was collected in EDTA containing vacutainers, packed in a sealed envelope and sent over postal mail to a central laboratory. At the laboratory, the EDTA samples were centrifuged for 10 minutes at 1200 g and plasma was stored at -80°C. Fatty acids were measured in plasma cholesteryl esters and plasma phospholipids (for the last 998 participants) by gas chromatography. In short, to isolate cholesteryl esters and phospholipids, lipids from EDTA plasma were dissolved and separated by solid phase extraction silica columns (Chrompack, Middelburg, The Netherlands). The fatty acids were identified by comparison with known standards (Nu-chek prep, Inc. Elysian, MN, USA). Fatty acids were expressed as mass percentages of total fatty acid methyl esters (g/100 g). A quality control plasma pool was analysed in duplicate in each run.

*Outcome ascertainment*: Incident diabetes during the trial phase was defined as either a self-reported physician diagnosis or use of antidiabetic medication (based on telephone interviews at 12 and 24 months or examinations at 20 months (midterm examination; n=800) or 40 months (final examination).

**Chin-Shan Community Cardiovascular Cohort (CCCC), Taiwan**^5,6^

*Cohort description*: CCCC began in 1990, following 1703 men and 1899 women aged 35 years old and above, homogenous in Chinese ethnicity, in Northern Taiwan for the study of cardiovascular diseases. The cohort was assembled from the registry data of the bureaucracy and the participants were recruited by house-to-house visits. The study was approved by the IRB in the National Taiwan University Hospital. Participants received baseline health examination at the community health center. We recruited the subjects by volunteer basis and respondent rates were up to 83%. In the survey, all of the study participants were individually interviewed from a structured questionnaire, for the information on socio-demographic characteristics, physical activity, smoking, alcohol drinking habits, dietary characteristics, personal and family histories of diseases and hospitalizations. With informed consent, the participants underwent physical examinations and laboratory tests. The examiners undertook training in the questionnaire collections and measures.

*Fatty acid measurement*: 10-mL tubes of EDTA-anticoagulated blood were collected, refrigerated on-site, and forwarded to the core laboratory of the National Taiwan University Hospital within 3 h. The blood was centrifuged at 800×g for 10 min, whereupon plasma was separated, dispensed into aliquots, and frozen at –70 ^o^C. All analyses of fatty acid content were performed by the same technician. After thawing the plasma, 0.5 mL samples were extracted and combined with 0.5 mL methanol followed by 1.0 mL chloroform under a nitrogen atmosphere. The lipid extract was then filtered to remove proteins and methyl esters were separated and measured using a 5890 gas chromatograph (Hewlett Packard, Avondale, PA) equipped with a 30 m-FFAT WCOT glass capillary column (J & W Scientific, Folsom, CA) and a flameionization detector. A total of 29 individual fatty acids were identified by comparing the retention times of peaks to the retention times of synthetic FA standards with known compositions (Supelco 37 Comp. FAME Mix, 47885-U; Bellefonte, PA, USA). The relative quantity of each FA (% of total FAs) was determined by integrating the area beneath the peak, and dividing the result by the total area for all FAs.
 *Outcome ascertainment*: During the follow-up from 1990 to 2000, incident diabetes was determined based on fasting glucose levels ≥7.0 mmol/l (≥126 mg/dL) or by the use of oral hypoglycaemic or insulin medication. The follow-up rate was 85.7%.

**Cardiovascular Health Study (CHS), the United States**^7–9^

*Cohort description*: The CHS is a population-based longitudinal study of risk factors for cardiovascular disease and stroke in adults 65 years of age or older, recruited at four field centers (Forsyth County, NC; Sacramento County, CA; Washington County, MD; Pittsburgh, PA). Overall, 5201 predominantly Caucasian individuals were recruited in 1989-1990 from random samples of Medicare eligibility lists, followed by an additional 687 African-Americans recruited in 1992-1993 (total n=5,888). Fatty acids were measured on samples collected in the third year of follow-up. Investigators of CHS can be found at <http://www.chs-nhlbi.org>.

*Fatty acid measurement*: Blood was drawn after a 12-hour fast and stored at -70^o^C. Plasma phospholipid fatty acids were measured at the Fred Hutchinson Cancer Research Center (Seattle, WA) using stored blood samples from 1992-1993. Total lipids were extracted from plasma using the methods of Folch.^10^ A one dimensional thin-layer chromatography was used to separate phospholipids from neutral lipids. Phospholipids fraction was directly trans-esterified using the Lepage and Roy method to prepare fatty acid methyl esters, and individual fatty acid methyl esters were separated using gas chromatography (Agilent 5890 Gas Chromatograph flame ionization detector, Agilent Technologies, Palo Alto, CA; fused silica capillary column SP-2560 [100m x 0.25mm, 0.2μm], Supelco Belefonte, PA; initial 160 degrees Celsius for 16 min, ramp 3 degrees Celsius/min to 240 degrees Celsius, hold 15 minutes). For this analysis, levels of each individual fatty acid are expressed as a weight percentage of total phospholipid fatty acids analyzed. CVs were <3% for most fatty acids.

*Outcome ascertainment*: Participants were followed by means of annual study clinic examinations with interim phone contacts for 10 y and telephone contacts every 6 mo thereafter. Medication use was assessed annually. Fasting glucose was measured at the study baseline (1992–1993) and in 1996–1997, 1998–1999, and 2005–2006; nonfasting glucose was measured in 1994–1995. Type 2 diabetes was defined as a single measure of fasting glucose concentration ≥126 mg/dL, nonfasting or 2-h postchallenge glucose concentration ≥200 mg/dL, or new use of an insulin or oral hypoglycemic medication.

**Framingham Heart Study (FHS), the United States** ^11–13^

*Cohort description*: FHS is a population based longitudinal study of families living in Framingham, Massachusetts. The offspring study was initiated in 1971 and consisted of a sample of 5,124 individuals, offspring of the original cohort and their spouses. Blood samples for fatty acid measurement and covariate data were collected during wave 8 of the study (2005-2008), and participants were followed till 2015.

*Fatty acid measurement*: The fatty acid composition of erythrocyte samples were analyzed by gas chromatography equipped with a SP 2560 capillary column after direct transesterification for 10 minutes in boron trifluoride/ methanol and hexane at 100 C as previously described.[^15^](#_ENREF_15) This technique generates fatty acids primarily from erythrocyte glycerophospholipids. Erythrocytes were isolated from blood drawn after a 10–12 h fast and frozen at −80 °C immediately after collection. All fatty acids present at >1% abundance had CVs of ≤7%.

*Outcome ascertainment*: Incident diabetes was defined as fasting glucose concentration ≥ 126 mg/dL, HBA1C ≥ 6.5 or new use of insulin or oral hypoglycemic medication ascertained during follow up examinations.

**Health Professionals’ Follow-up Study (HPFS), the United States**^14,15^

*Cohort description*: The Health Professionals Follow-up Study (HPFS) started in 1986, with 51,529 male health professionals, who were 40 – 75 years of age at recruitment in 1986. Blood samples were collected from HPFS participants in 1994. For this study we utilized previously measured fatty acid concentrations in stored blood used for nested case-control studies of incident cardiovascular diseases. Subjects were free of cardiovascular diseases, cancer and diabetes at the time of blood sampling.

*Fatty acid measurement*: Blood samples were sent to the lab with an ice pack via overnight courier and the majority of the samples arrived within 24 hours. Upon arrival, samples were centrifuged and divided into aliquots for plasma, white blood cell, and red blood cells, and stored in liquid nitrogen freezers at ≤-130°C. Fatty acid concentrations were measured in stored total plasma and erythrocyte samples using gas-liquid chromatography. Concentrations of individual circulating fatty acids were expressed as a percentage of total fatty acids either in plasma or erythrocyte membranes The average intra-assay CV were 10% for erythrocyte linoleic acid, 10% for erythrocyte arachidonic acid, 7% for plasma linoleic acid, and 10% for plasma arachidonic acid.

*Outcome ascertainment*: Incident cases of diabetes are identified by self-reports on mail questionnaires and confirmed by supplementary information collected about the diagnosis using the following criteria from the National Diabetes Data Group (NDDG) up until 1998: (1) manifestation of classic symptoms such as excessive thirst, polyuria, weight loss and hunger, in conjunction with elevated fasting glucose ≥140 mg/dL or non-fasting glucose levels ≥200 mg/dL (2) asymptomatic but elevated plasma glucose in two separate occasions or abnormal glucose tolerance test results and (3) receiving any hypoglycemic treatment for diabetes. After 1998 a fasting glucose concentration ≥126 mg/dL was adopted per the new diagnostic criteria of the American Diabetes Association (ADA). Medical records were obtained for a subset of the subjects diagnosed with diabetes to validate the information obtained by the supplemental questionnaire. This supplemental questionnaire has been validated as a confirmation tool for diabetes diagnosis with high reliability (>98% of cases confirmed for those who provided records).

**InterAct consortium (InterAct), eight European countries**^16,17^

*Cohort description*: The InterAct consortium is a case-cohort study derived from 340,234 people with 3.99 million person-years of follow-up (1991-2007) in eight countries of the EPIC study (France, Italy, Spain, the United Kingdom, the Netherlands, Germany, Sweden, and Denmark). In total, 12403 cases of type 2 diabetes were verified. From the European Prospective Investigations into Cancer and Nutrition cohort, 16,835 people with baseline plasma samples were randomly selected as a sub-cohort. After exclusions for prevalent diabetes and uncertain diabetes status, 16154 individuals remained in the sub-cohort, including 778 with incident type 2 diabetes during follow-up. From this case-cohort of 27,779 participants, 27,296 adults were available for analysis (12,132 cases of type 2 diabetes and 15,919 subcohort participants including 755 incident cases of type 2 diabetes within the sub-cohort). When providing results to the FORCE Consortium, single InterAct estimates were derived by conducting country-specific Prentice-weighted Cox proportional hazard analysis and pooling country-specific estimates by random-effects meta-analysis – a standard approach in InterAct.

*Fatty acid measurement*: Fatty acids were profiled at the Medical Research Council Human Nutrition Research (Cambridge, UK); profiling involved analysis of plasma samples stored at baseline at −196°C (or −150°C in Denmark). The assay methods were previously described and included hydrolysis and methylation to convert phospholipid fatty acids into more volatile fatty acid methyl esters and separation of the different fatty acids by gas chromatography (J&W HP-88, 30 m length, 0.25 mm internal diameter [Agilent Technologies, CA, USA]) equipped with flame ionisation detection (7890N GC [Agilent Technologies]). Samples from people with type 2 diabetes and subcohort participants were processed in random order by centre, and laboratory staff were masked to all participant characteristics by the use of anonymised aliquots. Thirty seven different fatty acids were identified with their retention times compared with those of commercial standards and expressed each level as percentage of total phospholipid fatty acids (mol%). Pentadecanoic acid (15:0) showed coefficient of variation 11.9%; palmitic acid (16:0), 1.6%; heptadecanoic acid (17:0), 4.2%; stearic acid (18:0), 2.0%; arachidic acid (20:0), 15.3%; behenic acid (22:0), 10.3%; tricosanoic acid (23:0), 18.9%; and lignoceric acid (24:0), 14.7%. We used human and equine plasma (Sera Laboratories International, West Sussex, UK) for quality control.

*Outcome ascertainment*: Incident type 2 diabetes was ascertained up until Dec 31, 2007, through a review of several sources of evidence: self-report, linkage to primary care registers, secondary care registers, medication use (drug registers), hospital admissions, and mortality data. No diabetes cases were ascertained solely by self-report and we sought further evidence for all cases with information about incident type 2 diabetes from fewer than two independent sources at a minimum, including a review of individual medical records in some centres. Cases in Denmark and Sweden were identified from local and national diabetes and pharmaceutical registers and were judged to be verified.

**Insulin Resistance Atherosclerosis Study (IRAS), the United States**^18,19^

*Cohort description*: IRAS is a multi-ethnic, multi-center observational cohort study. Participants were recruited at 4 clinical centers between October 1992 and April 1994. Ethnicity was determined by self-report. Two of the clinical centers (Los Angeles, CA, and Oakland, CA) were assigned to recruit African American and non-Hispanic white participants. In these centers, individuals were sampled from the members of a nonprofit health maintenance organization. In the other 2 clinical centers (San Luis Valley, CA, and San Antonio, TX), Hispanic and non-Hispanic white participants were recruited from ongoing population-based epidemiologic studies. All participants provided informed consent as approved by their respective field center's institutional review board. For the purpose of fatty acids, a subcohort of individuals without baseline diabetes was chosen (n = 749). Participants with missing fatty acids (n = 30) were excluded from the present investigation.

*Fatty acid measurement*: Fatty acids were measured in fasting plasma samples, utilizing a targeted, quantitative gas chromatography approach. Briefly, the lipids from plasma were extracted in the presence of authentic internal standards using chloroform:methanol (2:1 v/v), the Folch method^10^. The total lipid extract was trans-esterified in 1% sulfuric acid in methanol in a sealed vial under a nitrogen atmosphere at 100°C for 45 min. The resulting samples were neutralized with 6% potassium carbonate and the fatty acid methyl esters (FAME) re-extracted with hexane and prepared for gas chromatography. Fatty acid methyl esters were separated and quantified by capillary gas chromatography (Agilent Technologies model 6890) equipped with a 30 m DB-88MS capillary column (Agilent Technologies) and a flame-ionization detector. Quantitative results were obtained by comparing each fatty acid to its internal standard control. All fatty acid concentrations analyzed here passed internal quality assurance and quality control processes. Each fatty acid was expressed as a percentage of the total fatty acid. Inter-assay CVs were 7% for linoleic acid and 5% for arachidonic acid.

*Outcome ascertainment*: Participants were followed up after a period of 5 years from study baseline 1992 – 1994 in 1997 – 1999. Fasting glucose, 2 – hour post challenge glucose, glycated hemoglobin, use of insulin or oral hypoglycemic medication was ascertained at that time by trained technicians. Type 2 diabetes was defined as 1) fasting glucose concentration ≥ 126 mg/dL, or 2) 2-hour post oral glucose concentration ≥ 200 mg/dL, or 3) new use of an insulin or oral hypoglycemic medication, or 4) Fasting or non-fasting HbA1C concentration ≥6.5%.

**Melbourne Collaborative Cohort Study (MCCS), Australia**^20,21^

*Cohort description*: MCCS is a prospective cohort study of 41,514 residents (17,045 men) of Melbourne, Australia aged between 27 and 75 years at baseline (99.3% were ages 40– 69 years). Italian and Greek migrants were deliberately recruited to extend the range of lifestyle exposures. Recruitment occurred between 1990 and 1994. Participants were recruited via the electoral rolls (registration to vote is compulsory for adults in Australia), advertisements, and community announcements in local media (e.g., television, radio, and newspapers). Comprehensive lists of Italian and Greek surnames also were used to target southern European migrants in the phone book and electoral rolls. Blood was collected into sodium-heparin vacutainers from all subjects. After collection, blood was centrifuged immediately and plasma stored in liquid nitrogen at -120°C. Following an average of 9.0 (SD 1.2) years, samples for participants in the sub-cohort were defrosted at room temperature. Thawed samples were vortexed rapidly for a few seconds, then spun at 1000 rpm (210 x g) for 10 min at 4°C on a “Heraeus Megafuge 2.0R” bench top centrifuge. Samples were kept on ice until they were aliquotted. The aliquotted samples were also kept on ice. Liquid nitrogen was added to the tubes, which were sealed quickly, then frozen immediately at -80°C until shipment to the laboratory in cryoboxes. All samples were handled under red light conditions.

*Fatty acid measurement*: Biomarker fatty acids were measured in plasma collected at recruitment in a sub-group of around 6900 participants based on a random cohort of around 4000 people and cases of cancer, cardiovascular diseases and diabetes: the number of diabetes cases was 490 for the current study, informing a crude 4-year risk of 1.1% (490/41514). Total lipids were extracted from plasma with chloroform/methanol (2:1 by volume). Lipid extracts were separated by thin-layer chromatography (TLC) into phospholipids, triglyceride and cholesteryl esters classes on silica gel plates (Silica gel 60H Merck Darmstadt Germany). The TLC solvent system was petroleum spirit/diethyl ether/glacial acetic acid (180:30:2, by volume). Lipid classes were visualized with fluorescein 5-isothiocyanate against TLC standard 18-5 (NuChek Prep Inc: Elysian, MN) All solvents contained the anti-oxidant butylated hydroxyl anisole at 0.005% (wt/vol). Phospholipid fractions were transesterified by methanolysis (1% H2SO4 in methanol) for 3 h at 70°C. After cooling, the resulting FAME were extracted with n-heptane and transferred into gas chromatography vials containing anhydrous Na2SO4. FAME were separated and quantified with a Hewlett-Packard 5880 gas-liquid chromatograph using a capillary column equipped with fame ionization detection and Hewlett-Packard Chem-Station data system. Separation was achieved on a 50m×0.33mm ID BPX-70 column (SGE, Melbourne, Australia). Helium was the carrier gas at a column flow rate of 35 cm/s. The inlet split ratio was set at 30 to 35 cm/s. The oven temperature at injection was set at 140°C and programmed to rise to 200°C at 5°C/min. The injector and dejector temperatures were set to 250°C and 300°C, respectively. FAME were identified by comparison of retention times to authentic lipid standards (NuChek Prep Inc: Elysian, MN). The between batch coefficients of variation were between 1% and 12%.

*Outcome ascertainment*: Approximately 4 years after baseline, the participants completed a mailed, self-administered questionnaire that covered diagnosis of diabetes.

**Metabolic Syndrome in Men (METSIM), Finland**^22,23^

*Cohort description*: The population-based METSIM study includes 10 197 Finnish men, aged from 45 to 73 y at the baseline study (2005-2010) and who were living in Kuopio or surrounding communities and were willing to participate in the study. Plasma FAs were measured in a random sample of 1364 men of the entire cohort.[^22^](#_ENREF_22)

*Fatty acid measurement*: lipids were extracted from plasma sample with chloroform–methanol (2:1) and lipid fractions were separated using an aminopropyl column. Fatty acids in lipid fractions were transmethylated with 14% boron trifluoride in methanol. Finally, fatty acid methyl esters were analysed using a 7890A gas chromatograph (Agilent Technologies, Wilmington, DE, USA) equipped with a 25 m NEFA phase column (Agilent Technologies). Cholesteryl nonadecanoate (Nu-Chek Prep, Elysian, MA, USA), trinonadecanoin and dinonadecanoyl phosphatidylcholine (Larodan Fine Chemicals, Malmo, Sweden) served as internal standards.

*Outcome ascertainment*: Type 2 diabetes diagnosis was based on an oral glucose tolerance test (fasting plasma glucose ≥126 mg/dL and/or 2 h plasma glucose ≥ 200 mg/dL), or on HbA1c measurements (≥6.5 %) at the follow-up visit, or on the National Drug Reimbursement registry data (drug treatment started for diabetes during the follow-up).

**Multiethnic Study of Atherosclerosis (MESA), the United States**^24–26^

*Cohort description*: MESA is a National Heart, Lung and Blood Institute-sponsored, population-based investigation of subclinical cardiovascular disease and its progression. A total of 6,814 individuals, aged 45 to 84 years, were recruited from six US communities (Baltimore City and County, MD; Chicago, IL; Forsyth County, NC; Los Angeles County, CA; New York, NY; and St. Paul, MN) between July 2000 and August 2002. Participants were excluded if they had physician-diagnosed cardiovascular disease prior to enrollment, including angina, myocardial infarction, heart failure, stroke or TIA, resuscitated cardiac arrest or a cardiovascular intervention (e.g., CABG, angioplasty, valve replacement, or pacemaker/defibrillator placement). Pre-specified recruitment plans identified four racial/ethnic groups (White European-American, African-American, Hispanic-American, and Chinese-American) for enrollment, with targeted oversampling of minority groups to enhance statistical power. Investigators of MESA can be found at [http://www.mesa-nhlbi.org](https://urldefense.proofpoint.com/v2/url?u=http-3A__www.mesa-2Dnhlbi.org_&d=DwMFaQ&c=z4vlxFg302Kzizd2vlT_VEPVguFLXmhFCOQ4GP8RFxI&r=Up8UmShy1X9MbBjl91WkgxzJcL7bKDqz0njxMP3OhqE&m=VFIk3jGXw4WsoC2HBmMdcStS68LmnXcmWJ-cBzOxxcU&s=Gz_p7M7mULNQ9lEGco8_qQXpaCa9CPOobblEutHaSE0&e=).

*Fatty acid measurement*: Phospholipid fatty acids were extracted and measured at the University of Minnestoa (Minneapolis, MN). Plasma was diluted in saline and lipids were extracted from with a mixture of chloroform:methanol, and cholesterol, triglycerides and phospholipid subclasses were separated on a silica thin-layer chromatography plate in a solvent mixture of petroleum ether, diethyl ether, and glacial acetic acid. The band of phospholipids was harvested for the formation of methyl esters. FAME prepared with 14% boron trifluoride in methanol, incubated at 80ºC for 90 minutes, and extracted with petroleum ether. The final product was dissolved in heptane and injected onto a capillary Varian CP7420 100-m column with a Hewlett Packard 5890 gas chromatograph (GC) equipped with a HP6890A autosampler. The GC is configured for a single capillary column with a flame ionization detector and interfaced with HP chemstation software. Separation of individual fatty acids was obtained over an 80-minute run. Individual fatty acid values are expressed as percentage of total fatty acids. Inter-assay CVs were less than 10%.

*Outcome ascertainment*: Participants were followed by means of bi-annual study clinic examinations with yearly interim phone contacts for 10 year Fasting glucose was measured at the study baseline (2000-2002) and in 2002-2004,2004-2005, 2005-2007, and 2010–2011. Diabetes was defined as fasting plasma glucose ≥126 mg/dL.

**Nurses’ Health Study (NHS), the United States**^14,15^

*Cohort description*: NHS was established in 1976 by recruiting 121,700 female nurses aged 30 to 55 who responded to a questionnaire with information related to their health, lifestyle practices and occurrence of chronic diseases. Blood samples were collected from NHS participants in 1989-1990. For this study we utilized previously measured fatty acid concentrations in stored blood used for nested case-control studies of incident cardiovascular diseases. Subjects were free of cardiovascular diseases, cancer and diabetes at the time of blood sampling.

*Fatty acid measurement*: Blood samples were sent to the lab with an ice pack via overnight courier and the majority of the samples arrived within 24 hours. Upon arrival, samples were centrifuged and divided into aliquots for plasma, white blood cell, and red blood cells, and stored in liquid nitrogen freezers at ≤-130°C. Fatty acid concentrations were measured in stored total plasma and erythrocyte samples using gas-liquid chromatography. Concentrations of individual circulating fatty acids were expressed as a percentage of total fatty acids either in plasma or erythrocyte membranes. CVs were 10% for linoleic acid and arachidonic acid for erythrocyte membrane, 7% for linoleic in plasma, and 10% for arachidonic acid in plasma

*Outcome ascertainment*: Incident cases of T2D are identified by self-reports on the mail questionnaires and confirmed by supplementary information collected about the diagnosis using the following criteria from the National Diabetes Data Group (NDDG) up until 1998: (1) manifestation of classic symptoms such as excessive thirst, polyuria, weight loss and hunger, in conjunction with elevated fasting glucose ≥140 mg/dL or non-fasting glucose levels ≥200 mg/dL (2) asymptomatic but elevated plasma glucose in two separate occasions or abnormal glucose tolerance test results and (3) receiving any hypoglycemic treatment for diabetes. After 1998 a fasting glucose concentration ≥126 mg/dL was adopted per the new diagnostic criteria of the American Diabetes Association (ADA). Medical records were obtained for a subset of the subjects diagnosed with diabetes to validate the information obtained by the supplemental questionnaire. This supplemental questionnaire has been validated as a confirmation tool for diabetes diagnosis with high reliability (>98% of cases confirmed for those who provided records).

**Prospective Investigation of the Vasculature in Uppsala Seniors (PIVUS), Sweden**^27–30^

*Cohort description*: PIVUS was initiated in 2001 as a research collaboration between the Department of Medicine and the University Hospital in Uppsala with the primary aim to evaluate the usefulness of different measurements of endothelial function and other techniques to evaluate vascular function. In June 2004 the last subject was included in the cohort resulting in 1016 subjects aged 70 being randomly selected from the general population in the town of Uppsala. Several secondary aims have also been added to this prospective cohort study and several academic groups have been engaged in the evaluation of this cohort from different aspects.

*Fatty acid measurement*: Fatty acid composition in cholesterol esters and phospholipids were measured by gas chromatography. Serum (0.5 mL) was mixed with 2.5 mL methanol, 5 mL chloroform (with 0.005% added butylated hydroxytoluene, BHT) and 7.5 mL NaH2PO4 (0.2 mol/l) and stored in 4°C over night for lipid extraction. The chloroform phase was then removed with a syringe and evaporated to dryness on a 30°C heating block using nitrogen gas. The lipid residue was dissolved in chloroform and the lipid fractions were separated by thin-layer chromatography (TLC); the adsorbent containing POPOP as fluorescent agent. The TLC-plates were eluted at room temperature with the solvent system petroleum ether/diethyl ether/acetic acid (81:18:1 by volume). The lipid fractions were visualized in UV light and the spots containing cholesterol esters and phospholipids were scraped off into vials and methylated at 60°C overnight after addition of 2 mL H2SO4 (5%) in methanol. The fatty acid methyl esters were extracted into 3 mL petroleum ether (0.005% BHT) after addition of 1.5 mL distilled water. The phases were separated after thorough mixing and centrifugation at 1500g for 10 minutes. The petroleum ether phase was pipetted off and the solvent was evaporated under nitrogen gas on a 30°C heating block. The fatty acid methyl esters were dissolved in 120 µL hexane and placed in vials. The fatty acid methyl esters were separated by gas-liquid chromatography on a 30-m glass capillary column coated with Thermo TR-FAME (Thermo Electron Corporation, USA) with helium gas as a carrier gas. An Agilent Technologies system consisting of model GLC 6890N, autosampler 7683 and Agilent ChemStation was used. The temperature was programmed to 150-260° C. The fatty acids were identified by comparing each peak´s retention time with fatty acid methyl ester standards Nu Check Prep (Elysian, MN, USA). Fatty acids are presented as the percent of total fatty acids analyzed in each compartment.

*Outcome ascertainment*: Diabetes incidence during follow-up was identified by medical records, repeated blood sampling and self-reports at follow-ups after 5 and 10 years. Type 2 diabetes was defined using one of four criteria: a) fasting blood glucose ≥110 mg/dL (corresponds to fasting plasma glucose ≥ 126mg/dL), b) self-reported diabetes, c) diabetes diagnosis reported in medical records, d) use of insulin or oral hypoglycemic agents.

**Three City Study (3C), France** ^31,32^

*Cohort description*: 3C study is an ongoing multicenter prospective cohort study of vascular risk factors for dementia which started in 1999-2000 and included 9,294 community dwellers in three French cities: Bordeaux (n=2,104), Dijon (n=4,931) and Montpellier (n=2,259). Individuals living in one of these cities, aged 65 years and over and not institutionalized were eligible for recruitment into the 3C study. The protocol of the 3C study has been approved by the Consultative Committee for the Protection of Persons participating in Biomedical Research of the Kremlin-Bicêtre University Hospital (Paris). All participants gave their written informed consent. The baseline data collection included socio-demographic and lifestyle characteristics, symptoms and complaints, main chronic conditions, medication use, neuropsychological testing, clinical examination including blood pressure measurement, electrocardiogram (ECG) and blood sampling. Four follow-up examinations were performed at 2, 4, 7, and 10 years after baseline. The present study is based on the 12 years of follow-up. Fatty acid composition of red blood cell membrane phospholipids were measured at baseline from fasting blood samples among 670 individuals from the Bordeaux and Montpellier centers.

*Fatty acid measurement*: Erythrocyte membrane phospholipid fatty acids were measured at the French Institute for fats and oils (ITERG). Total lipids from red blood cell membranes were extracted by using the method of Peuchant et al.^33^ A one dimensional thin-layer chromatography was used to separate total phospholipids of red blood cells from neutral lipids. Total fatty acids of the red blood cell phospholipid fraction were methylated according to the procedure of Morrison and Smith,[^26^](#_ENREF_26) to obtain fatty acid methyl esters. Individual fatty acid methyl esters were separated using a gas chromatograph (Focus GC, Thermo Scientific, France) equipped with a flam ionization detector and a split injector. A fused silica capillary column (BPX 70, 60m x 0.25mm internal diameter, 0.25mm film; Phenomenex, Germany) was used with H2 as the carrier gas (inlet pressure: 1 bar). The column temperature was programmed to increase from 150 to 200°C at 1.5°C/min for 25 min, and then from 200 to 225°C at 20°C/min and was held at 225°C until the completion of the analysis (20 min). The injection port and detector were maintained at 250 and 280°C, respectively. Data were integrated using the ChromQuest Software (Thermo Scientific). Individual fatty acid methyl esters were identified by comparing their retention times with those of authentic standards eluted in the same conditions (Sigma Chemical Co., Saint Quentin Fallavier, France). The results are expressed as a weight percentage of total fatty acids. The laboratory CV's were The laboratory CV's were 0.5% for linoleic acid and 0.8% for arachidonic acid.

*Outcome ascertainment*: Participants were followed up at 2, 4,7,10,12 years post baseline. Type 2 diabetes was self -reported or defined by use of insulin or oral hypoglycemic medication.

**Uppsala Longitudinal Study of Adult Men (ULSAM), Sweden**^27,28,30,34^

*Cohort description*: ULSAM is a community-based cohort of men living in Uppsala county, Sweden. The origin of this longitudinal study was the "Uppsala Primary Preventive Study", carried out between September 1970 and September 1973. The study comprised all men living in the County of Uppsala born between 1920 and 1924 selected from the register of County Council. All men (n=2841) were invited for the investigation, 81.7% (n=2322) participated. The mean age at this baseline examination was 49.6 (SD +/- 0.6), hence this starting cohort was referred to as ULSAM-50. After this baseline examination, all men were invited to participate in follow-up investigations at the ages 70, 82 and 88. Between the age 50 and 70, 422 had died and 219 had moved out of the Uppsala region. Of the 1681 men invited, 460 did not participate in this follow up, leaving 1221 men who participated (response rate of 73%) aged around 70. The men were invited by a letter, which also explained the aim of the examination. They received the letter 7-10 days prior to the examination. Those born at the beginning of the year were called first. Six individuals were called every weekday except for the vacation period in Sweden between June 25 and August 15. A second invitation letter was sent at the end of the examination of each age class to those who had not come after the first invitation. The screening examination program included a medical questionnaire and interview, blood and urine sampling, blood pressure and anthropometric measurements, intravenous glucose tolerance test, ECG recording, chest X-ray and pure tone audiometry. At the baseline exam, fatty acid composition was assessed in serum cholesterol, whereas at the second exam 20 years later, fatty acids were measured in both cholesterol esters and adipose tissue. Dodecapenta and dodecahepta acids were measured only in adipose tissue lipids.

*Fatty acid measurement*: Adipose tissue fatty acid composition was initially analyzed in a random subsample of 318 men. In December 2008, 535 new samples were analyzed for adipose tissue fatty acid composition. Subcutaneous adipose tissue was collected with biopsy as described by Hirsch et al.^35^ and Beynen et al.^36^. The subject lay face down and the biopsy was taken with a needle coupled to a vacuum tube from the upper, outer quadrant of the buttocks. The sample was collected in the connector between the needle and the tube, and stored at -70°C in the connector for some weeks until analysis. Prior to the fatty acid analysis the biopsy was weighed and homogenized. The fatty acid compositions of the subcutaneous adipose tissue were analyzed as described in detail by Carlson^37^ and Boberg et al.^38^. An extraction with chloroform in the presence of methanol, butylated hydroxytoluene, and NaH2PO4 was conducted over night, and evaporated under nitrogen. The dry extracts were dissolved in a few drops of chloroform and applicated on thin liquid chromatography plates for separation of the lipids in a solvent system consisting of petroleum ether:diethyl ether:acetic acid (81:18:1, by volume) (Boberg 1966). The lipid fractions were visualized in UV light and scraped off separately. The lipid esters were trans methylated in warm, acidic environment overnight. The methylesters were extracted with petroleum ether and deionized water, and the solvent was evaporated under nitrogen. The fatty acid methyl esters were dissolved in hexane and separated by gas-liquid chromatography (GLC). The Hewlett Packard GLC system used for the analyses was consisted a GC 5890, automatic sampler 7671A, integrator 3392A, and 25 m Quadrex Fused Silica capillary column OV-351, with helium as the carrying gas. The temperature program used during the separation of the fatty acid methyl esters was 130-220°C. The fatty acids were identified by comparison of the retention times of separation was controlled by Nu Check Prep GLC reference standard GLC-68A.

*Outcome ascertainment*: Incident diabetes during follow-up was identified using the Swedish Hospital Discharge and Cause of Death registers, and supplemented with ULSAM clinical assessments that occurred at throughout follow-up. All participants were followed regarding incidence of diabetes until December 31, 2011. Using registry data, diabetes was identified according to International Classification of Disease 9^th^ (ICD-9) and 10^th^ revision (ICD-10), codes 250 and E10-E14, respectively. Diabetes prevalence at baseline and subsequent ULSAM clinical assessments were determined as fasting blood glucose ≥110 mg/dL (corresponds to fasting plasma glucose ≥ 126 mg/dL) or fasting plasma glucose ≥126 mg/dL, or the use of glucose-lowering medication.

**Women’s Health Initiatives Memory Study (WHI), the United States**^39–41^

*Cohort description*: WHI was established to examine the effects of postmenopausal hormone therapy on cognitive function in women aged 65-80 years. Recruitment began in June 1995. Of 3200 eligible women free of probable dementia enrolled in the WHI, 2947 (92.1%) were enrolled in WHIMS.  Diabetes status was last assessed in August 2009. Investigators of WHIMS can be found at [http://www.whi.org/researchers/](https://urldefense.proofpoint.com/v2/url?u=http-3A__www.whi.org_researchers_Documents-2520-2520Write-2520a-2520Paper_WHI-2520Investigator-2520Long-2520List.pdf&d=DwMGaQ&c=z4vlxFg302Kzizd2vlT_VEPVguFLXmhFCOQ4GP8RFxI&r=Up8UmShy1X9MbBjl91WkgxzJcL7bKDqz0njxMP3OhqE&m=DcTHSiNMEqmcyeUXON5rZ0i8muwwkw4is618zieDfe8&s=gk2hdWheTfV_eWAFVR6p3jx-PgUNL83C0yH8lbmx9Dc&e=).

*Fatty acid measurement*: The fatty acid composition of RBC samples were analyzed by gas chromatography equipped with a SP 2560 capillary column after direct transesterification for 10 minutes in boron trifluoride/ methanol and hexane at 100 C as previously described. This technique generates fatty acids primarily from RBC glycerophospholipids. During the aliquoting phase, the RBC samples were stored improperly at -20°C for a period of approximately 2 weeks, causing oxidative degeneration of the PUFAs before measurement. The original FA levels were estimated with multiple imputations using independent data on fatty acid degradation and length of time the samples were exposed to -20°C.^41^ All fatty acids present at >1% abundance had CVs of ≤6.5%. Genotyping was conducted using the Human Omni Express Exome-8v1_B, with imputation using the 1000 genomes reference panel.

*Outcome ascertainment*: Incident diabetes was defined as a positive answer to the question (asked annually) regarding “newly prescribed treatment for diabetes with pills or insulin shots.” The date of diabetes onset was assigned as the midpoint between the dates between the survey when diabetes was self-reported and the previous survey.

# References

1. Harris TB, Launer LJ, Eiriksdottir G, et al. Age, Gene/Environment Susceptibility-Reykjavik Study: multidisciplinary applied phenomics. *Am J Epidemiol*. 2007;165(9):1076–87.

2. Harris TB, Song X, Reinders I, et al. Plasma phospholipid fatty acids and fish-oil consumption in relation to osteoporotic fracture risk in older adults: the Age, Gene/Environment Susceptibility Study. *Am J Clin Nutr*. 2015;101(5):947–955.

3. Kromhout D, Giltay EJ, Geleijnse JM, Alpha Omega Trial Group. n-3 fatty acids and cardiovascular events after myocardial infarction. *N Engl J Med*. 2010;363(21):2015–26.

4. Geleijnse JM, Giltay EJ, Schouten EG, et al. Effect of low doses of n-3 fatty acids on cardiovascular diseases in 4,837 post-myocardial infarction patients: design and baseline characteristics of the Alpha Omega Trial. *Am Heart J*. 2010;159(4):539–546.e2.

5. Chien K, Cai T, Hsu H, et al. A prediction model for type 2 diabetes risk among Chinese people. *Diabetologia*. 2009;52(3):443–450.

6. Chien K-L, Lin H-J, Hsu H-C, et al. Comparison of predictive performance of various fatty acids for the risk of cardiovascular disease events and all-cause deaths in a community-based cohort. *Atherosclerosis*. 2013;230(1):140–7.

7. Fried LP, Borhani NO, Enright P, et al. The Cardiovascular Health Study: design and rationale. *Ann Epidemiol*. 1991;1(3):263–76.

8. Tell GS, Fried LP, Hermanson B, Manolio TA, Newman AB, Borhani NO. Recruitment of adults 65 years and older as participants in the Cardiovascular Health Study. *Ann Epidemiol*. 1993;3(4):358–66.

9. Mozaffarian D, Cao H, King IB, et al. Trans-palmitoleic acid, metabolic risk factors, and new-onset diabetes in U.S. adults: a cohort study. *Ann Intern Med*. 2010;153(12):790–9.

10. Sloane-Stanley JFMLGH. A simple method for the isolation and purification of total lipides from animal tissues. *J Lipid Res*. 1957;226(3):497–509.

11. Harris WS, Pottala J V, Vasan RS, Larson MG, Robins SJ. Changes in erythrocyte membrane trans and marine fatty acids between 1999 and 2006 in older Americans. *J Nutr*. 2012;142(7):1297–303.

12. Wilson PW, Meigs JB, Sullivan L, et al. Prediction of incident diabetes mellitus in middle-aged adults: the Framingham Offspring Study. *Arch Intern Med*. 2007;167(10):1068–1074.

13. Feinleib M, Kannel WB, Garrison RJ, McNamara PM, Castelli WP. The Framingham Offspring Study. Design and Preliminary Data. *Prev Med*. 1975;4(4):518–525.

14. Malik VS, Chiuve SE, Campos H, et al. Circulating Very-Long-Chain Saturated Fatty Acids and Incident Coronary Heart Disease in US Men and Women. *Circulation*. 2015;132(4):260–8.

15. Yakoob MY, Shi P, Willett WC, et al. Circulating Biomarkers of Dairy Fat and Risk of Incident Diabetes Mellitus Among US Men and Women in Two Large Prospective Cohorts. *Circulation*. 2016;133(17):1645–1654.

16. The InterAct Consortium. Design and cohort description of the InterAct Project: an examination of the interaction of genetic and lifestyle factors on the incidence of type 2 diabetes in the EPIC Study. *Diabetologia*. 2011;54(9):2272–2282.

17. Forouhi NG, Koulman A, Sharp SJ, et al. Differences in the prospective association between individual plasma phospholipid saturated fatty acids and incident type 2 diabetes: the EPIC-InterAct case-cohort study. *Lancet Diab Endocrinol*. 2014;2(10):810–818.

18. Wagenknecht LE, Mayer EJ, Rewers M, et al. The insulin resistance atherosclerosis study (IRAS) objectives, design, and recruitment results. *Ann Epidemiol*. 1995;5(6):464–72.

19. Santaren ID, Watkins SM, Liese AD, et al. Serum pentadecanoic acid (15:0), a short-term marker of dairy food intake, is inversely associated with incident type 2 diabetes and its underlying disorders. *Am J Clin Nutr*. 2014;100(6):1532–1540.

20. Hodge AM, Simpson JA, Gibson RA, et al. Plasma phospholipid fatty acid composition as a biomarker of habitual dietary fat intake in an ethnically diverse cohort. *Nutr Metab Cardiovasc Dis*. 2007;17(6):415–26.

21. Hodge AM, English DR, O’Dea K, et al. Plasma phospholipid and dietary fatty acids as predictors of type 2 diabetes: interpreting the role of linoleic acid. *Am J Clin Nutr*. 2007;86(1):189–97.

22. Stančáková A, Javorský M, Kuulasmaa T, Haffner SM, Kuusisto J, Laakso M. Changes in Insulin Sensitivity and Insulin Release in Relation to Glycemia and Glucose Tolerance in 6,414 Finnish Men. *Diabetes*. 2009;58(5):1212–1221.

23. Lankinen M a., Stančáková A, Uusitupa M, et al. Plasma fatty acids as predictors of glycaemia and type 2 diabetes. *Diabetologia*. 2015;58(11):2533–2544.

24. Bild DE, Bluemke DA, Burke GL, et al. Multi-Ethnic Study of Atherosclerosis: Objectives and Design. *Am J Epidemiol*. 2002;156(9):871–881.

25. Mozaffarian D, de Oliveira Otto MC, Lemaitre RN, et al. trans-Palmitoleic acid, other dairy fat biomarkers, and incident diabetes: the Multi-Ethnic Study of Atherosclerosis (MESA). *Am J Clin Nutr*. 2013;97(4):854–61.

26. Cao J, Schwichtenberg KA, Hanson NQ, Tsai MY. Incorporation and Clearance of Omega-3 Fatty Acids in Erythrocyte Membranes and Plasma Phospholipids. *Clin Chem*. 2006;52(12):2265–2272.

27. Hagström E, Kilander L, Nylander R, et al. Plasma parathyroid hormone is associated with vascular dementia and cerebral hyperintensities in two community-based cohorts. *J Clin Endocrinol Metab*. 2014;99(11):4181–9.

28. Huang X, Sjögren P, Ärnlöv J, et al. Serum fatty acid patterns, insulin sensitivity and the metabolic syndrome in individuals with chronic kidney disease. *J Intern Med*. 2014;275(1):71–83.

29. Rosqvist F, Bjermo H, Kullberg J, et al. Fatty acid composition in serum cholesterol esters and phospholipids is linked to visceral and subcutaneous adipose tissue content in elderly individuals: a cross-sectional study. *Lipids Health Dis*. 2017;16(1):68.

30. Fall T, Salihovic S, Brandmaier S, et al. Non-targeted metabolomics combined with genetic analyses identifies bile acid synthesis and phospholipid metabolism as being associated with incident type 2 diabetes. *Diabetologia*. 2016;59(10):2114–24.

31. 3C Study Group. Vascular factors and risk of dementia: design of the Three-City Study and baseline characteristics of the study population. *Neuroepidemiol*. 2003;22(6):316–25.

32. Samieri C, Maillard P, Crivello F, et al. Plasma long-chain omega-3 fatty acids and atrophy of the medial temporal lobe. *Neurology*. 2012;79(7):642–50.

33. Peuchant E, Wolff R, Salles C, Jensen R. One-step extraction of human erythrocyte lipids allowing rapid determination of fatty acid composition. *Anal Biochem*. 1989;181(2):341–4.

34. Hedstrand H. A study of middle-aged men with particular reference to risk factors for cardiovascular disease. *Upsala J Med Sci*. 1975;19:1–61.

35. Hirsch J, Farquhar JW, Ahrens EH, Peterson ML, Stoffel W. Studies of adipose tissue in man. A microtechnic for sampling and analysis. *Am J Clin Nutr*. 1960;8:499–511.

36. Beynen AC, Katan MB. Rapid sampling and long-term storage of subcutaneous adipose-tissue biopsies for determination of fatty acid composition. *Am J Clin Nutr*. 1985;42(2):317–22.

37. Carlson LA. Determination of serum triglycerides. *J Atheroscler Res*. 1963;3(4):334–336.

38. Vahlquist C, Berne B, Boberg M, Michaëlsson G, Vessby B. The fatty-acid spectrum in plasma and adipose tissue in patients with psoriasis. *Arch Dermatol Res*. 1985;278(2):114–119.

39. Espeland MA, Rapp SR, Shumaker S a, et al. Conjugated equine estrogens and global cognitive function in postmenopausal women: Women’s Health Initiative Memory Study. *JAMA*. 2004;291(24):2959–68.

40. Harris WS, Luo J, Pottala J V., Margolis KL, Espeland MA, Robinson JG. Red Blood Cell Fatty Acids and Incident Diabetes Mellitus in the Women’s Health Initiative Memory Study. *PLoS One*. 2016;11(2):e0147894.

41. Pottala J V, Espeland MA, Polreis J, Robinson J, Harris WS. Correcting the effects of -20 °C storage and aliquot size on erythrocyte fatty acid content in the Women’s Health Initiative. *Lipids*. 2012;47(9):835–46.
