# Supplementary material for: Fatty acid biomarkers of dairy fat consumption and incidence of type 2 diabetes: A pooled analysis of prospective cohort studies
Source: PLoS Med. 2018 Oct 10;15(10):e1002670. doi: 10.1371/journal.pmed.1002670 (PMC6179183; doi:10.1371/journal.pmed.1002670)
Supplement: S2 Text — (DOCX) [file pmed.1002670.s008.docx]

S2 Text. Study Protocol
 **Fatty Acid Biomarkers of Dairy Consumption and Incident Diabetes: a Meta-Analysis of Prospective Cohort Studies**

Lead authors: Fumiaki Imamura, Nita G Forouhi, Amanda Fretts, Rozenn N Lemaitre, Dariush Mozaffarian on behalf of the CHARGE Fatty Acids and Outcome Pooling Project

Correspondence:

Fumiaki Imamura, PhD MS

University of Cambridge, MRC Epidemiology Unit

Cambridge Biomedical Campus, Cambridge, CB2 0QQ, UK

fumiaki.imamura@mrc-epid.cam.ac.uk

Background:

Habitual consumption of dairy products is a potentially important modifiable factor for chronic diseases. Type 2 diabetes (T2D) is a major cause of morbidity and mortality and one of noncommunicable diseases for which consumption of dairy products may play a role in prevention. A recent meta-analysis has shown consumption of dairy products to be inversely associated with incident type 2 diabetes (T2D).^1^ Higher consumption of total dairy products by 400 g/day was related to 7% (95% confidence interval=1% to 13%) lower risk of T2D. This inverse association is important for a dietary recommendation of dairy products for prevention of T2D. However, the magnitude of the association may be underestimated because of random misclassification in estimation of dietary consumption assessed by self-reported dietary methods in cohort studies.^1^ To strengthen the evidence for the inverse association, therefore, objective measurement of the dietary exposure is of great interest.

Circulating levels of odd-chain fatty acids (15:0 and 17:0) and *trans*-palmitoleic acid (*t*16:1n7) have been known to be objective biomarkers of consumption of dairy products.^2–5^ The prospective associations of relative concentrations of these biomarkers with incident T2D were examined in several studies in the US, the UK, Sweden, Germany, and Australia.^6–11^ Findings have been inconsistent. A significant inverse association of 15:0 with T2D was observed in the studies in Germany, Sweden, and Australia, whereas two US studies and the UK study showed no significant association. The inverse association of 17:0 with T2D was observed in the British and Swedish studies, but not in two US studies and the German study. Additionally, the study in Germany found a significant inverse association of sum of 15:0 and 17:0 with incident T2D, although each was not significantly associated when evaluated separately. Finally, *t*16:1n7 have been examined only in the two US studies, and found to be associated with lower risk of T2D consistently.

Different conclusions have been drawn for the associations of circulating levels of 15:0 and 17:0 with incident T2D. The heterogeneity may partly reflect imprecise estimates of the association due to small sample sizes as well as differences in population demographics, study design, and laboratory methods. Therefore, meta-analysis to relate these fatty acid biomarkers to incident T2D, including analysis of heterogeneity, will help better understand the association of consumption of dairy products with incident T2D.

To examine the association with a large sample size and examine sources of the heterogeneity, we propose a study to test the hypothesis that circulating levels of 15:0, 17:0, and *trans*-16:1n7 (if measured) and their combination (15:0+17:0+*t*16:1n7 or 15:0+17:0) are associated with incident T2D, using the available information of CHARGE Fatty Acids Phenotype working group.

**PROJECT AIM:**

To examine the association of the following fatty acids with incident T2D:

(a) 15:0

(b) 17:0

(c) *t*16:1n7

(d) 15:0+17:0+*t*16:1n7 (or two of them if one is not available)

**METHODS:**

Sample:

- Adults (aged 18+ years) from the CHARGE Fatty Acid Phenotype working group with measures of circulating 15:0, 17:0, and *t*16:1n7

Both cohort studies and case-control studies with available data on any of circulating 15:0, 17:0, and *t*16:1n7, and incident diabetes data are eligible to participate in the project.

Exclusions:

- Participants <18 years of age
- Prevalent diabetes
- No data on any of circulating 15:0, 17:0, and *t*16:1n7

Dependent Variable:

- incident diabetes (as defined by the cohort)

Independent variables:

1. biomarker 15:0;
2. biomarker 17:0;
3. biomarker *t*16:1n7

If more than one fatty acid biomarker is available (e.g., the study has data available for both plasma phospholipid fatty acids and cholesterol ester fatty acids), please provide analysis results for each of the biomarkers.

For the purposes of this analysis, the relationship of each fatty acid of interest with diabetes will be assessed:

1. using study-specific quintiles (primary)
2. continuously (% total fatty acids per 1 SD increment; for nested case-control studies, we will use the SD of the control group) (secondary)

Analysis Plan for Each Participating Study (study-specific analyses):

Baseline descriptive information will be collected (see Information to Be Collected section below for details on variables of interest). Additionally, we will assess the correlations of 15:0, 17:0 and *t*16:1n7 with each other.

The association of each individual fatty acid of interest (15:0, 17:0, *t*16:1n7, and sum of these) with incident diabetes will be assessed both categorically (quintiles) and continuously (% total fatty acids per 1 SD increment) using multiple Cox regression models (for cohort studies) or conditional logistic regression (for case-control studies). Follow-up time will be calculated from baseline (time of fatty acid measurement) to date of development of incident diabetes, death from any cause, or loss to follow-up; participants will be censored at the time of diabetes diagnosis, death, or loss to follow-up.

We will examine the association of each fatty acid of interest with incident diabetes using results from three regression models that adjust for different sets of covariates. The primary model will adjust for potential confounders, including age, sex, field site (if necessary), socio-demographic variables (race, education, and occupation), health behaviors (physical activity, smoking, and alcohol use), family history of diabetes, menopausal status, exogenous hormone use, prevalent hypertension (treated or self-reported), prevalent dyslipidemia (treated or self-reported), prevalent coronary heart disease, and self-reported health status. A second model will additionally adjust for BMI and waist circumference. A third model (exploratory model) will further adjust for circulating 16:0 and triglycerides, known potential mediators or confounders in the Nurses’ Health Study and the Health Professionals’ Follow-up Study (unpublished).

We will also examine potential interaction of age (modeled continuously), sex, race, and BMI (modeled continuously) with each plasma phospholipid saturated fatty acid of interest (modeled continuously) on risk of incident diabetes. To evaluate interaction, a multiplicative interaction term for each factor of interest will be included in a model that also adjusts for age, sex, field site (if necessary), socio-demographic variables (race, education, and occupation), health behaviors (physical activity, smoking, and alcohol use), family history of diabetes, menopausal status, exogenous hormone use, prevalent hypertension (treated or self-reported), prevalent dyslipidemia (treated or self-reported), prevalent coronary heart disease, and self-reported health status; and BMI (for models that assess the interaction of each fatty acid with BMI).

Analysis Plan in Pooling Results:

Descriptive statistics will be tabulated across studies.

Meta-analyses will examine the relationship of each circulating fatty acid (15:0, 17:0, *t*16:1n7, and sum of these) with incident diabetes using inverse-variance-weighted fixed-effects models. We will also examine potential interaction of each circulating (15:0, 17:0, *t*16:1n7, and sum of these) with age, sex, race, and BMI on risk of diabetes by meta-analyzing the multiplicative interaction terms for each age, sex, race, or BMI with each fatty acid of interest on risk of incident diabetes.

Additionally, we will test for potential heterogeneity by country, fasting state, lipid fraction, and study design, by meta-regression and by stratified meta-analysis.

**INFORMATION TO BE REQUESTED:**

**(I) Descriptive information:**

We will request the following study-specific information. If the study is a nested case-control study, please provide descriptive information for the controls only.

- Baseline (year)
- End of follow-up (year)
- Total number of participants available for analysis
- Total number of participants excluded (i.e.,<18 years of age, prevalent diabetes)
- Total number of incident cases of diabetes
- Total number of person-years (if a nested case-control study, person-years of the source population will be requested)
- Circulating 15:0 (mean, median, SD, range)
- Circulating 17:0 (mean, median, SD, range)
- Circulating *t*16:1n7 (mean, median, SD, range)
- Sum of 15:0, 17:0, and *t*16:1n7 (mean, median, SD, range)

If multiple lipid fractions were assessed, statistics of each fraction will be obtained.

- Sex (% female)
- Age (mean±SD)
- Race (study specific)
- Smoking (% never, %former, % current)
- Alcohol intake (drinks/day: mean±SD or grams/day: mean±SD)
- BMI (kg/m^2^) and waist circumference (cm) (mean±SD each)
- Prevalent hypertension, dyslipidemia, heart disease (% each)
- Menopausal status and hormone use among women (% in women for each)
- Family history of diabetes (%)
- Socioeconomic variables (e.g. categories of education and occupation, as defined by each study)
- Physical activity (hours/week: mean±SD or kcal/week: mean±SD or otherwise defined by the study)
- HbA1c (% of hemoglobin)
- Fasting glucose (mg/dL) (mean±SD)
- Fasting insulin (IU/mL) (mean±SD)
- HOMA-IR (mean±SD)
- HOMA-β (mean±SD)
- Triglycerides (mg/dL) (mean±SD)
- HDL cholesterol (mg/dL) (mean±SD)

(II) Methodological information

- Laboratory methods and relevant citation information for fatty acids assessment, including the tissue fraction of fatty acids measured.
- Fatty acid fractions
- Total number of fatty acids measured
- Laboratory methods and relevant citation information for fasting glucose assessment

(III) Correlations between fatty acids

- Pearson Correlation matrix of 15:0, 17:0, *t*16:1n7, and sum of these.

For a nested case-control studies, please provide correlation matrix for controls

(IV) Main results (regression coefficients and robust standard errors, SE)

1. Analysis of 15:0, 17:0, *t*16:1n7, and sum of these fatty acids with incident diabetes (each variable modeled separately)

Model 1: adjusted for age, sex, field site (if necessary), race, education, occupation, physical activity, smoking, alcohol use, family history of diabetes, menopausal status, exogenous hormone use, prevalent hypertension (treated or self-reported), prevalent dyslipidemia (treated or self-reported), prevalent coronary heart disease, and self-reported health status

Model 2: adjusted for all model 1 covariates, as well as BMI and waist circumference

Model 3: adjusted for all model 2 covariates, as well as triglycerides and circulating 16:0

For quintile analyses, please provide:

- regression coefficients + robust SE for each quintile (referent group=lowest quintile)
- number of diabetes cases in each quintile
- total n in each quintile
- total person-years in each quintile
- median level of circulating in each quintile

For continuous analyses (per SD), please provide:

- regression coefficients + robust SE

A spreadsheet (excel file) that details all requested information will be prepared and distributed to each participating cohort.

(V) Results for potential interaction

We will assess the interaction of each circulating levels of 15:0, 17:0, and *t*16:1n7, and sum of these with

(1) age (modeled continuously)

(2) sex (male, female)

(3) race (using cohort-specific dummy variables)

(4) BMI (modeled continuously)

on risk of diabetes to examine potential heterogeneity.

(1) Analyses include a multiplicative (cross-product) interaction term for each of 15:0, 17:0, and *t*16:1n7, and sum of these fatty acids with age, sex, race or BMI (modeled separately) in a model also adjusted for age, sex, field site (if necessary), socio-demographic variables (race, education, and occupation), health behaviors (physical activity, smoking, and alcohol use), family history of diabetes, menopausal status, exogenous hormone use, prevalent hypertension (treated or self-reported), prevalent dyslipidemia (treated or self-reported), prevalent coronary heart disease, self-reported health status, BMI and waist-circumference.

For each interaction analysis, please provide:

- regression coefficient + robust SE the cross-product interaction term

A spreadsheet (excel file) that details all requested information for the interaction analyses will be prepared and distributed to each participating cohort. If a significant interaction is observed by pooling study-specific interaction terms in fixed-effect meta-analyses, stratified analyses of the factor of interest will be requested after post-hoc decision of cut points for stratification.

SUMMARY

Associations of dairy products with incident diabetes are of scientific and public health interest. To add robust knowledge on the topic, we will assess the association of circulating levels of fatty acid biomarkers of dairy consumption with incident diabetes in the FORCE consortium.

**REFERENCES**

1. Aune D, Norat T, Romundstad P, Vatten LJ. Dairy products and the risk of type 2 diabetes: a systematic review and dose-response meta-analysis of cohort studies. *The American journal of clinical nutrition*. 2013;98(4):1066–83.

2. Brevik A, Veierod MB, Drevon CA, Andersen LF. Evaluation of the odd fatty acids 15:0 and 17:0 in serum and adipose tissue as markers of intake of milk and dairy fat. *Euro J Clin Nutr*. 2005.

3. Wolk A, Vessby B, Ljung H, Barrefors P. Evaluation of a biological marker of dairy fat intake. *Am J Clin Nutr*. 1998;68(2):291–295.

4. De Oliveira Otto MC, Nettleton JA, Lemaitre RN, et al. Biomarkers of dairy fatty acids and risk of cardiovascular disease in the Multi-ethnic Study of Atherosclerosis. *J Am Heart Assoc*. 2013;2(4):e000092.

5. Hodson L, Eyles HC, McLachlan KJ, Bell ML, Green TJ, Skeaff CM. Plasma and erythrocyte fatty acids reflect intakes of saturated and n-6 PUFA within a similar time frame. *The Journal of nutrition*. 2014;144(1):33–41.

6. Krachler B, Norberg M, Eriksson JW, et al. Fatty acid profile of the erythrocyte membrane preceding development of Type 2 diabetes mellitus. *Nutrition, Metabolism and Cardiovascular Diseases*. 2008;18(7):503–510.

7. Patel PS, Sharp SJ, Jansen E, et al. Fatty acids measured in plasma and erythrocyte-membrane phospholipids and derived by food-frequency questionnaire and the risk of new-onset type 2 diabetes: a pilot study in the European Prospective Investigation into Cancer and Nutrition (EPIC)–Norfolk. *Am J Clin Nutr*. 2010;92(5):1214–1222.

8. Kröger J, Zietemann V, Enzenbach C, et al. Erythrocyte membrane phospholipid fatty acids, desaturase activity, and dietary fatty acids in relation to risk of type 2 diabetes in the European Prospective Investigation into Cancer and Nutrition (EPIC) - Potsdam Study. *Am J Clin Nutr*. 2010;93(1):127–142.

9. Mozaffarian D, Cao H, King IB, et al. Trans-palmitoleic acid, metabolic risk factors, and new-onset diabetes in U.S. adults: a cohort study. *Annals of internal medicine*. 2010;153(12):790–9.

10. Mozaffarian D, de Oliveira Otto MC, Lemaitre RN, et al. trans-Palmitoleic acid, other dairy fat biomarkers, and incident diabetes: the Multi-Ethnic Study of Atherosclerosis (MESA). *Am J Clin Nutr*. 2013;97(4):854–61.

11. Hodge AM, English DR, O’Dea K, et al. Plasma phospholipid and dietary fatty acids as predictors of type 2 diabetes: interpreting the role of linoleic acid. *Am J Clin Nutr*. 2007;86(1):189–97.
