# Supplementary material for: Fatty acid biomarkers of dairy fat consumption and incidence of type 2 diabetes: A pooled analysis of prospective cohort studies
Source: PLoS Med. 2018 Oct 10;15(10):e1002670. doi: 10.1371/journal.pmed.1002670 (PMC6179183; doi:10.1371/journal.pmed.1002670)
Supplement: S1 Checklist — (DOCX) [file pmed.1002670.s009.docx]

**S3 Text. PRISMA Checklist

The authors’ note**: While PRISMA is disseminated for a systematic review and a meta-analysis, we selected this checklist because our consortium project summarising evidence from multiple cohorts is suited to the aim of PRISMA. Of note, all the analyses from participating cohorts were standardised and the submitted work was not based on published literature but based on cohorts participating the FORCE consortium (see details at <http://force.nutrition.tufts.edu/about>). Therefore check-items for publication bias and bias due to heterogeneity in analytic methods were considered not relevant to this work.

| **Section/topic** | **#** | **Checklist item** | **Reported on page # (or a location in a sub-section)** |
| --- | --- | --- | --- |
| **TITLE** | | |  |
| Title | 1 | Identify the report as a systematic review, meta-analysis, or both. | Identifiable as a pooling project of the FORCE consortium. |
| **ABSTRACT** | | |  |
| Structured summary | 2 | Provide a structured summary including, as applicable: background; objectives; data sources; study eligibility criteria, participants, and interventions; study appraisal and synthesis methods; results; limitations; conclusions and implications of key findings; systematic review registration number. | Abstract |
| **INTRODUCTION** | | |  |
| Rationale | 3 | Describe the rationale for the review in the context of what is already known. | Introduction:  The first paragraph highlights the importance of studying dairy consumption and its relationship with noncommunicable diseases; and the second paragraph states the limitation of research on dietary consumption of dairy products, including difficulty of measuring dairy consumption with a self-reported instrument. The last paragraph indicates scarcity of research on fatty acid biomarkers and their relationships with incident type 2 diabetes. |
| Objectives | 4 | Provide an explicit statement of questions being addressed with reference to participants, interventions, comparisons, outcomes, and study design (PICOS). | Introduction, the last paragraph: “To address these limitations and provide new evidence on relationships between these biomarkers and T2D, we conducted a pooling project to test the hypothesis that higher concentrations of 15:0, 17:0, and t16:1n7 would be associated with lower incident T2D, evaluating adults free from T2D in prospective cohorts participating the Fatty Acids and Outcomes Research Consortium (FORCE).” |
| **METHODS** | | |  |
| Protocol and registration | 5 | Indicate if a review protocol exists, if and where it can be accessed (e.g., Web address), and, if available, provide registration information including registration number. | Methods, the second paragraph:  “A standardized analysis protocol (S2 Text) was developed and provided to each participating cohort.” |
| Eligibility criteria | 6 | Specify study characteristics (e.g., PICOS, length of follow-up) and report characteristics (e.g., years considered, language, publication status) used as criteria for eligibility, giving rationale. | Methods, the second paragraph: “adults aged 18 years or older, not with diabetes, and with data on fatty acids and incident T2D” |
| Information sources | 7 | Describe all information sources (e.g., databases with dates of coverage, contact with study authors to identify additional studies) in the search and date last searched. | Information from 16 cohorts has been provided in the supplementary materials (S2 Text). |
| Search | 8 | Present full electronic search strategy for at least one database, including any limits used, such that it could be repeated. | Not applicable to this consortium project. |
| Study selection | 9 | State the process for selecting studies (i.e., screening, eligibility, included in systematic review, and, if applicable, included in the meta-analysis). | Methods, the first paragraph: “included 16 prospective studies (cohorts, nested case-control studies, or nested case-cohort studies) that met the inclusion criteria and agreed to participate: recruitment of adults aged 18 years or over and including participants free from diabetes at the time of fatty acid assessment; assessment of circulating or adipose 15:0, 17:0 or *t*16:1n7; and follow up for incident T2D (S1 Text)..” |
| Data collection process | 10 | Describe method of data extraction from reports (e.g., piloted forms, independently, in duplicate) and any processes for obtaining and confirming data from investigators. | Methods, the second paragraph:  “A standardized analysis protocol (S2 Text) was developed and provided to each participating cohort. It included inclusion criteria (adults aged 18 years or older, not with diabetes, and with data on fatty acids and incident T2D), exposures, covariates, effect modifiers, outcomes, and longitudinal analyses.” |
| Data items | 11 | List and define all variables for which data were sought (e.g., PICOS, funding sources) and any assumptions and simplifications made. | Methods, the second and third paragraphs, Table 1, and supplementary materials. |
| Risk of bias in individual studies | 12 | Describe methods used for assessing risk of bias of individual studies (including specification of whether this was done at the study or outcome level), and how this information is to be used in any data synthesis. | Methods, the subsection of ‘Meta-analysis’, corresponding to standard cohort analyses varying degrees of covariate adjustment; tests for interactions; and assessment of heterogeneity by cohorts. |
| Summary measures | 13 | State the principal summary measures (e.g., risk ratio, difference in means). | Methods, the subsection of ‘Meta-analysis’: “Study-specific regression coefficients and measures of precision (standard errors) from each of continuous and categorical terms were pooled with an inverse-variance weighted meta-analysis to estimate summary relative risks (RRs) per the 10th to 90th percentile range and quintile categories.” |
| Synthesis of results | 14 | Describe the methods of handling data and combining results of studies, if done, including measures of consistency (e.g., I^2^) for each meta-analysis. | Methods, the subsection of ‘Meta-analysis’: Between-study heterogeneity was expressed as I-squared. |
| Risk of bias across studies | 15 | Specify any assessment of risk of bias that may affect the cumulative evidence (e.g., publication bias, selective reporting within studies). | Methods, the subsection of ‘Meta-analysis’: Influence analysis and assessment of heterogeneity and interactions (tests for homogeneity of associations by measured covariates). |
| Additional analyses | 16 | Describe methods of additional analyses (e.g., sensitivity or subgroup analyses, meta-regression), if done, indicating which were pre-specified. | Methods, the subsection of ‘Meta-analysis’, including analysis to obtain sex-specific estimates. |
| **RESULTS** | | |  |
| Study selection | 17 | Give numbers of studies screened, assessed for eligibility, and included in the review, with reasons for exclusions at each stage, ideally with a flow diagram. | Not applicable to this consortium work. |
| Study characteristics | 18 | For each study, present characteristics for which data were extracted (e.g., study size, PICOS, follow-up period) and provide the citations. | Table 1.Several descriptive statistics have not been presented because different cohorts have different types of information varied by unit or categories or missing information which did not influence our planned analysis because the information was collected for descriptive purpose. |
| Risk of bias within studies | 19 | Present data on risk of bias of each study and, if available, any outcome level assessment (see item 12). | Fig 2 and Supporting Information show point estimates from analyses with different degrees of adjustment for covariates (not necessarily the source of bias), but the series of harmonised analyses indicate the degree of bias due to confounding. Also results by study characteristics are presented in S1 Table. |
| Results of individual studies | 20 | For all outcomes considered (benefits or harms), present, for each study: (a) simple summary data for each intervention group (b) effect estimates and confidence intervals, ideally with a forest plot. | Fig 2 and Fig 3. |
| Synthesis of results | 21 | Present results of each meta-analysis done, including confidence intervals and measures of consistency. | Fig 2, Fig 3 and S Figs; and the latter half of the Results section presented with Fig 2 and Fig 3. |
| Risk of bias across studies | 22 | Present results of any assessment of risk of bias across studies (see Item 15). | The last paragraph of Results |
| Additional analysis | 23 | Give results of additional analyses, if done (e.g., sensitivity or subgroup analyses, meta-regression [see Item 16]). |  |
| **DISCUSSION** | | |  |
| Summary of evidence | 24 | Summarize the main findings including the strength of evidence for each main outcome; consider their relevance to key groups (e.g., healthcare providers, users, and policy makers). | Main results have been summarised in the first paragraph of the Discussion. |
| Limitations | 25 | Discuss limitations at study and outcome level (e.g., risk of bias), and at review-level (e.g., incomplete retrieval of identified research, reporting bias). | Discussion, the second paragraph and the second last paragraph, mainly about the limited ability for the biomarkers to distinguish exposures to different types of dairy fat consumption based on the fatty acid biomarkers; residual confounding; and measurement errors. |
| Conclusions | 26 | Provide a general interpretation of the results in the context of other evidence, and implications for future research. | The paragraph before Acknowledgements |
| **FUNDING** | | |  |
| Funding | 27 | Describe sources of funding for the systematic review and other support (e.g., supply of data); role of funders for the systematic review. | Financial Disclosure statement |

*From:*  Moher D, Liberati A, Tetzlaff J, Altman DG, The PRISMA Group (2009). Preferred Reporting Items for Systematic Reviews and Meta-Analyses: The PRISMA Statement. PLoS Med 6(6): e1000097. doi:10.1371/journal.pmed1000097

For more information, visit: **www.prisma-statement.org**.
